# Supplementary material for: Tracking extinction risk trends and patterns in a mega-diverse country: A Red List Index for birds in Colombia
Source: PLoS One. 2020 Jan 27;15(1):e0227381. doi: 10.1371/journal.pone.0227381 (PMC6984723; doi:10.1371/journal.pone.0227381)
Supplement: S1 Table — There were no species that underwent genuine changes in status in lowland dry forest, paramo, coastal waters, Caribbean Coast and Ocean, Darién, SNSM, San Andrés and Providencia, nor among nocturnal raptors. (PDF) [file pone.0227381.s001.pdf]

Table S1. Disaggregated Red List Index values for 2002 and 2016 for different regions, ecosystems and species groups. There were no species that underwent genuine changes in status in coastal waters, Caribbean Coast and Ocean, Darién, SNSM, San Andrés and Providencia, lowland dry forest, paramo, nor among nocturnal raptors.

**Region**	**2002**	**2016**
Pacific	0.987	0.984
Pacific Ocean	0.978	0.974
Caribbean Sea	0.968	0.968
Andes	0.954	0.953
Caribbean	0.984	0.983
Amazon	0.998	0.998
Orinoquia	0.997	0.996
Darién highlands	0.963	0.962
Sierra Nevada de Santa Marta	0.976	0.976
San Andrés & Providencia	0.964	0.964

**Ecosystem**	**2002**	**2016**
Lowland rainforest	0.986	0.985
Lowland dry forest	0.971	0.971
Sub-Andean forest	0.957	0.957
High Andean forest	0.945	0.946
Paramo	0.938	0.938
Mangrove	0.971	0.966
Savanna	0.978	0.977
Freshwater	0.950	0.946
Coastal waters	0.957	0.957

**Species group**	**2002**	**2016**
Gamebirds	0.876	0.869
Diurnal raptors	0.953	0.947
Nocturnal raptors	0.985	0.985
Forest raptors	0.920	0.907
Hummingbirds	0.937	0.950
Parrots	0.889	0.893
Oscine passerines	0.953	0.953
Suboscine passerines	0.970	0.970
Large frugivores	0.909	0.905
Terrestrial forest insectivores	0.923	0.920
Colombia	0.955	0.954
