# Supplementary material for: Tracking extinction risk trends and patterns in a mega-diverse country: A Red List Index for birds in Colombia
Source: PLoS One. 2020 Jan 27;15(1):e0227381. doi: 10.1371/journal.pone.0227381 (PMC6984723; doi:10.1371/journal.pone.0227381)
Supplement: S2 Table — Species are classified by regions, ecosystems and groups of conservation concern. The latter include Trochilidae (hummingbirds), Tytonidae and Strigidae (nocturnal raptors), Psittacidae (parrots) and Suboscines and Oscines passerines. Abbreviations: L. rain-forest = lowland rainforest; Sub-A. forest = sub-Andean forest; High A. forest = high-Andean forest; L. dry forest = lowland dry forest; Fwt. = Freshwater ecosystems; C.wt = coastal and pelagic waters. P. Ocean = Pacific Ocean; C. Sea = Caribbean Sea; SNSM = Sierra Nevada de Santa Marta; Sa&Pr = San Andrés & Providencia islands. T. forest insectiv. = terrestrial forest insectivores; Large Frugiv. = large frugivores. Taxonomy following South American Classification Committee (SACC), 2018. National and global categories according to Renjifo et al. 2002, Renjifo et al. 2016 and BirdLife International 2018 respectively. (PDF) [file pone.0227381.s002.pdf]

Table S2. Data used to calculate disaggregated indices. Species are classified by regions, ecosystems and groups of conservation concern. The latter include Trochilidae (hummingbirds), Tytonidae and Strigidae (nocturnal raptors), Psittacidae (parrots), Suboscines and Oscines passerines. Abbreviations: L. rain-forest= lowland rainforest; Sub-A. forest= sub-Andean forest; High A. forest= high-Andean forest; L. dry forest= lowland dry forest; Fwt.= Freshwater ecosystems; C.wt= coastal and pelagic waters. P. Ocean= Pacific Ocean; C. Sea= Caribbean Sea; SNSM= Sierra Nevada de Santa Marta; Sa&Pr= San Andrés & Providencia islands. T. forest insectiv.= terrestrial forest insectivores; Large Frugiv.= large frugivores. Taxonomy following South American Classification Committee (SACC), 2018. National and global categories according to Renjifo et al 2002, Renjifo et al 2016 and BirdLife International 2018 respectively.

				ECOSYSTEMS									REGIONS										GROUPS CONSERVATION CONCERN			
Family and species	National category 2002	National category 2016	Global category 2018	L. rain-forest	Sub-A. forest	High A. forest	L. dry forest	Par-amo	Man-grove	Sav-anna	Fwt.	C.wt	Pacific	P. Ocean	Andes	Caribbean	C. Sea	Amazon	Orinoquia	SNSM	Darién highlands	Sa&Pr.	Game-birds	T. forest insectiv.	Large Frugiv.	Forest Raptors
**Tinamidae**																										
Nothocercus julius	LC	LC	LC		x	x									x								x		x	
Nothocercus bonapartei	LC	LC	LC		x	x									x								x		x	
Tinamus tao	LC	LC	VU	x	x								x		x				x	x			x		x	
Tinamus osgoodi	EN	EN	VU		x										x								x		x	
Tinamus major	LC	LC	NT	x									x		x	x		x	x	x			x		x	
Tinamus guttatus	LC	LC	NT	x														x					x		x	
Crypturellus berlepschi	LC	LC	LC	x									x										x		x	
Crypturellus cinereus	LC	LC	LC	x						x								x	x				x		x	
Crypturellus soui	LC	LC	LC	x	x		x						x		x	x		x	x	x	x		x		x	
Crypturellus obsoletus	CR	CR	LC		x										x								x		x	
Crypturellus undulatus	LC	LC	LC	x														x					x		x	
Crypturellus duidae	LC	LC	NT	x														x	x				x		x	
Crypturellus erythropus	LC	LC	LC	x			x									x				x			x		x	
Crypturellus kerriae	VU	VU	VU	x									x										x		x	
Crypturellus variegatus	LC	LC	LC	x														x					x		x	
Crypturellus casiquiare	LC	LC	LC	x														x					x		x	
**Anhimidae**																										
Anhima cornuta	LC	LC	LC								x					x		x	x				x			
Chauna chavaria	VU	VU	NT								x					x							x			
**Anatidae**																										
Dendrocygna bicolor	LC	LC	LC						x		x					x	x		x				x			
Dendrocygna viduata	LC	LC	LC						x		x					x	x		x				x			
Dendrocygna autumnalis	LC	LC	LC						x		x		x	x		x	x	x	x				x			
Oressochen jubatus	NT	VU	NT								x							x	x				x			
Cairina moschata	LC	LC	LC								x		x			x		x	x				x			
Sarkidiornis melanotos	EN	EN	LC								x					x							x			
Amazonetta brasiliensis	LC	LC	LC								x								x				x			
Merganetta armata	LC	LC	LC								x				x								x			
Anas andium	LC	LC	LC								x				x								x			
Anas acuta	LC	LC	LC								x				x	x							x			
Anas cyanoptera	EN	EN	LC								x				x								x			
Anas bahamensis	NT	NT	LC						x		x	x		x		x	x						x			
Anas discors	LC	LC	LC								x		x		x	x		x	x			x	x			
Anas georgica	VU	VU	LC								x				x								x			
Anas clypeata	LC	LC	LC								x					x			x				x			
Netta erythrophthalma	CR	CR	LC								x				x	x							x			
Nomonyx dominicus	LC	LC	LC								x		x			x		x					x			
Oxyura jamaicensis	EN	EN	LC								x				x								x			
**Cracidae**																										
Chamaepetes goudotii	LC	LC	LC		x	x									x					x			x		x	
Penelope argyrotis	LC	LC	LC		x	x									x					x			x		x	
Penelope ortoni	VU	VU	EN	x	x								x		x								x		x	
Penelope montagnii	LC	LC	LC		x	x									x								x		x	
Penelope jacquacu	LC	LC	LC	x														x	x				x		x	
Penelope purpurascens	LC	LC	LC	x	x		x						x		x	x				x			x		x	
Penelope perspicax	EN	EN	EN	x	x										x								x		x	
Pipile cumanensis	LC	LC	LC	x														x	x				x		x	
Aburria aburri	LC	LC	NT	x	x										x					x			x		x	
Ortalis cinereiceps	LC	LC	LC	x	x								x		x								x		x	
Ortalis garrula	LC	LC	LC	x	x		x		x							x	x			x			x		x	
Ortalis ruficauda	LC	LC	LC	x			x			x						x			x	x			x		x	
Ortalis erythroptera	NT	NT	VU	x									x										x		x	

Family and species	National category 2002	National category 2016	Global category 2018	ECOSYSTEMS: L. rain-forest	Sub-A. forest	High A. forest	L. dry forest	Par-amo	Man-grove	Sav-anna	Fwt.	C.wt	REGIONS: Pacific	P. Ocean	Andes	Caribbean	C. Sea	Amazon	Orinoquia	SNSM	Darién highlands	Sa&Pr.	GROUPS CONSERVATION CONCERN: Game-birds	T. forest insectiv.	Large Frugiv.	Forest Raptors
Ortalis columbiana	LC	LC	LC	x	x		x								x	x							x		x	
Ortalis guttata	LC	LC	LC	x														x	x				x		x	
Nothocrax urumutum	LC	LC	LC	x														x	x				x		x	
Crax rubra	NT	VU	VU	x									x								x		x		x	
Crax alberti	CR	CR	CR	x											x	x				x			x		x	
Crax daubentoni	VU	EN	NT	x	x										x	x			x				x		x	
Crax alector	LC	LC	VU	x	x										x			x	x				x		x	
Crax globulosa	EN	EN	EN	x														x					x		x	
Mitu tomentosum	LC	LC	NT	x														x	x				x		x	
Mitu salvini	LC	LC	LC	x														x					x		x	
Mitu tuberosum	LC	LC	LC	x														x					x		x	
Pauxi pauxi	VU	EN	EN		x										x								x		x	
**Odontophoridae**																										
Rhynchortyx cinctus	LC	LC	LC	x	x								x		x						x		x		x	
Colinus cristatus	LC	LC	LC				x			x					x	x			x	x			x		x	
Odontophorus gujanensis	LC	LC	NT	x	x										x	x		x			x		x		x	
Odontophorus atrifrons	NT	NT	VU		x	x									x					x			x		x	
Odontophorus erythrops	LC	LC	LC	x	x								x		x	x							x		x	
Odontophorus hyperythrus	LC	LC	NT		x	x									x								x		x	
Odontophorus melanonotus	EN	EN	VU		x										x								x		x	
Odontophorus speciosus	LC	LC	NT	x	x										x			x					x		x	
Odontophorus dialeucos	EN	EN	VU		x																x		x		x	
Odontophorus strophium	EN	EN	VU		x										x								x		x	
**Phoenicopteridae**																										
Phoenicopterus ruber	EN	EN	LC									x					x									
**Podicipedidae**																										
Tachybaptus dominicus	LC	LC	LC								x				x	x		x								
Podilymbus podiceps	LC	LC	LC								x		x		x	x										
Podiceps andinus	EX	EX	EX								x				x											
Podiceps occipitalis	EN	CR	NT								x				x											
**Columbidae**																										
Patagioenas leucocephala	NT	NT	NT				x		x								x					x	x		x	
Patagioenas speciosa	LC	LC	LC	x	x					x			x		x	x		x	x	x			x		x	
Patagioenas corensis	LC	LC	LC				x									x				x			x		x	
Patagioenas fasciata	LC	LC	LC		x	x									x					x			x		x	
Patagioenas cayennensis	LC	LC	LC	x	x		x		x	x			x	x	x	x	x	x	x	x			x		x	
Patagioenas plumbea	LC	LC	LC	x	x								x		x	x		x					x		x	
Patagioenas subvinacea	LC	LC	VU	x	x	x							x		x	x		x	x		x		x		x	
Patagioenas nigrirostris	LC	LC	LC		x																x		x		x	
Patagioenas goodsoni	LC	LC	LC	x									x										x		x	
Geotrygon purpurata	LC	LC	EN	x									x										x		x	
Geotrygon saphirina	LC	LC	LC	x														x					x		x	
Geotrygon montana	LC	LC	LC	x	x								x		x	x		x	x	x			x		x	
Geotrygon violacea	LC	LC	LC	x	x										x					x	x		x			
Leptotrygon veraguensis	LC	LC	LC	x									x			x							x			
Leptotila verreauxi	LC	LC	LC	x	x	x	x			x			x		x	x		x	x	x			x			
Leptotila cassinii	LC	LC	LC	x									x			x							x			
Leptotila jamaicensis	CR	CR	LC				x															x	x		x	
Leptotila rufaxilla	LC	LC	LC	x						x								x	x				x			
Leptotila conoveri	VU	VU	EN		x										x								x			
Leptotila pallida	LC	LC	LC	x									x										x			
Zentrygon frenata	LC	LC	LC		x	x									x								x		x	
Zentrygon linearis	LC	LC	LC	x	x	x									x					x			x		x	
Zentrygon goldmani	VU	VU	NT		x																x		x			
Zenaida auriculata	LC	LC	LC							x			x		x	x			x	x			x			
Columbina passerina	LC	LC	LC				x			x					x	x		x	x	x		x	x			
Columbina minuta	LC	LC	LC				x			x			x		x	x		x	x				x			

Family and species				ECOSYSTEMS									REGIONS										GROUPS CONSERVATION CONCERN			
	National category 2002	National category 2016	Global category 2018	L. rain-forest	Sub-A. forest	High A. forest	L. dry forest	Par-amo	Man-grove	Sav-anna	Fwt.	C.wt	Pacific	P. Ocean	Andes	Caribbean	C. Sea	Amazon	Orinoquia	SNSM	Darién highlands	Sa&Pr.	Game-birds	T. forest insectiv.	Large Frugiv.	Forest Raptors
Columbina talpacoti	LC	LC	LC		x					x			x		x	x		x	x	x			x			
Columbina buckleyi	LC	LC	LC	x									x										x			
Columbina squammata	LC	LC	LC				x			x						x			x				x			
Columbina picui	LC	LC	LC	x														x					x			
Columbina cruziana	LC	LC	LC	x									x										x			
Claravis pretiosa	LC	LC	LC	x	x		x			x			x		x	x		x	x	x			x			
Claravis mondetoura	LC	LC	LC		x	x									x					x			x			
Metriopelia melanoptera	LC	LC	LC			x		x							x								x			
**Cuculidae**																										
Crotophaga major	LC	LC	LC	x	x				x				x	x	x	x	x	x	x	x						
Crotophaga ani	LC	LC	LC		x	x				x			x		x	x		x	x	x	x	x				
Crotophaga sulcirostris	LC	LC	LC				x			x			x		x	x			x	x						
Tapera naevia	LC	LC	LC	x	x					x			x		x	x		x	x	x						
Dromococcyx phasianellus	LC	LC	LC	x	x		x								x			x		x						
Dromococcyx pavoninus	LC	LC	LC	x	x										x											
Neomorphus geoffroyi	LC	LC	VU	x									x			x		x						x		
Neomorphus radiolosus	EN	EN	EN	x	x								x		x									x		
Neomorphus pucheranii	LC	LC	LC	x														x						x		
Coccycua minuta	LC	LC	LC	x	x		x			x			x		x	x		x	x							
Coccycua pumila	LC	LC	LC	x	x		x			x			x		x	x			x	x						
Piaya cayana	LC	LC	LC	x	x	x	x			x			x		x	x		x	x	x	x					
Piaya melanogaster	LC	LC	LC	x														x	x							
Coccyzus melacoryphus	LC	LC	LC	x	x				x						x	x	x	x	x							
Coccyzus americanus	LC	LC	LC	x	x	x	x			x			x		x	x		x	x	x	x	x				
Coccyzus erythropthalmus	LC	LC	LC	x	x	x	x						x		x	x				x	x	x				
Coccyzus lansbergi	LC	LC	LC	x	x		x						x			x				x	x					
**Steatornithidae**																										
Steatornis caripensis	LC	LC	LC	x	x	x		x					x		x			x	x	x					x	
**Nyctibiidae**																										
Nyctibius grandis	LC	LC	LC	x	x		x			x			x		x	x		x	x							
Nyctibius aethereus	LC	LC	LC	x									x					x	x							
Nyctibius griseus	LC	LC	LC	x	x	x	x						x		x	x		x	x	x						
Nyctibius maculosus	LC	LC	LC		x	x									x											
Nyctibius bracteatus	LC	LC	LC	x														x								
**Caprimulgidae**																										
Chordeiles nacunda	LC	LC	LC							x						x		x	x							
Chordeiles pusillus	LC	LC	LC							x								x	x							
Chordeiles rupestris	LC	LC	LC								x							x								
Chordeiles acutipennis	LC	LC	LC							x			x		x	x			x	x		x				
Chordeiles minor	LC	LC	LC							x			x		x	x		x	x	x		x				
Lurocalis semitorquatus	LC	LC	LC	x	x								x		x	x		x	x	x						
Lurocalis rufiventris	LC	LC	LC		x	x									x											
Nyctiprogne leucopyga	LC	LC	LC								x							x	x							
Nyctipolus nigrescens	LC	LC	LC	x						x								x	x							
Systellura longirostris	LC	LC	LC					x		x					x					x						
Nyctidromus albicollis	LC	LC	LC	x	x		x			x			x		x	x		x	x	x						
Uropsalis segmentata	LC	LC	LC			x		x							x											
Uropsalis lyra	LC	LC	LC		x										x											
Setopagis heterura	LC	LC	LC				x			x						x			x							
Hydropsalis cayennensis	LC	LC	LC		x		x			x			x		x	x			x							
Hydropsalis maculicaudus	LC	LC	LC							x			x		x	x			x							
Hydropsalis climacocerca	LC	LC	LC								x							x	x							
Nyctiphrynus rosenbergi	LC	LC	NT	x	x								x		x											
Nyctiphrynus ocellatus	LC	LC	LC	x														x								
Antrostomus carolinensis	LC	LC	NT	x	x		x								x	x										
Antrostomus rufus	LC	LC	LC	x			x						x			x			x	x						
**Apodidae**																										

Family and species	National category 2002	National category 2016	Global category 2018	ECOSYSTEMS									REGIONS										GROUPS CONSERVATION CONCERN			
				L. rain-forest	Sub-A. forest	High A. forest	L. dry forest	Par-amo	Man-grove	Sav-anna	Fwt.	C.wt	Pacific	P. Ocean	Andes	Caribbean	C. Sea	Amazon	Orinoquia	SNSM	Darién highlands	Sa&Pr.	Game-birds	T. forest insectiv.	Large Frugiv.	Forest Raptors
Cypseloides cherriei	DD	DD	DD		x										x											
Cypseloides cryptus	LC	LC	LC		x										x											
Cypseloides lemosi	DD	DD	LC	x	x		x								x											
Streptoprocne rutila	LC	LC	LC	x	x	x							x		x	x				x						
Streptoprocne zonaris	LC	LC	LC	x	x	x	x	x		x			x		x	x		x	x	x						
Chaetura spinicaudus	LC	LC	LC	x	x								x			x				x						
Chaetura cinereiventris	LC	LC	LC	x	x					x			x		x	x		x	x	x						
Chaetura pelagica	LC	LC	VU	x	x	x	x			x			x		x	x		x	x		x	x				
Chaetura chapmani	LC	LC	LC		x										x						x					
Chaetura viridipennis	LC	LC	N/A*	x														x								
Chaetura meridionalis	LC	LC	LC	x			x									x										
Chaetura brachyura	LC	LC	LC	x	x		x						x		x	x		x	x	x						
Aeronautes montivagus	LC	LC	LC	x	x										x			x		x						
Tachornis furcata	DD	DD	LC	x												x										
Tachornis squamata	LC	LC	LC	x						x								x	x							
Panyptila cayennensis	LC	LC	LC	x	x								x		x	x		x	x	x						
**Trochilidae (hummingbirds)**																										
Topaza pyra	LC	LC	LC	x														x								
Florisuga mellivora	LC	LC	LC	x	x		x						x		x	x		x	x	x	x					
Eutoxeres aquila	LC	LC	LC	x	x								x		x	x					x					
Eutoxeres condamini	LC	LC	LC	x														x								
Glaucis aeneus	LC	LC	LC	x	x								x		x											
Glaucis hirsutus	LC	LC	LC	x	x								x		x	x		x	x	x						
Threnetes ruckeri	LC	LC	LC	x	x								x		x	x				x						
Threnetes leucurus	LC	LC	LC	x	x										x			x								
Phaethornis rupurumii	LC	LC	LC	x															x							
Phaethornis atrimentalis	LC	LC	LC	x														x	x							
Phaethornis striigularis	LC	LC	LC	x	x								x		x	x				x						
Phaethornis griseogularis	LC	LC	LC	x	x										x											
Phaethornis ruber	LC	LC	LC	x														x	x							
Phaethornis augusti	LC	LC	LC	x	x		x								x					x						
Phaethornis anthophilus	LC	LC	LC	x	x										x	x				x						
Phaethornis hispidus	LC	LC	LC	x														x	x							
Phaethornis yaruqui	LC	LC	LC	x	x								x		x											
Phaethornis guy	LC	LC	LC	x	x										x						x					
Phaethornis syrmatophorus	LC	LC	LC	x	x	x									x											
Phaethornis bourcieri	LC	LC	LC	x														x	x							
Phaethornis longirostris	LC	LC	LC	x	x								x		x	x				x						
Phaethornis malaris	LC	LC	LC	x														x	x							
Doryfera ludovicae	LC	LC	LC		x	x									x											
Doryfera johannae	LC	LC	LC	x	x										x			x								
Schistes geoffroyi	LC	LC	LC	x	x	x									x											
Colibri delphinae	LC	LC	LC	x	x	x									x	x				x	x					
Colibri thalassinus	LC	LC	LC		x	x									x					x						
Colibri coruscans	LC	LC	LC		x	x		x							x					x						
Androdon aequatorialis	LC	LC	LC	x	x								x		x						x					
Heliothryx barroti	LC	LC	LC	x									x			x										
Heliothryx auritus	LC	LC	LC	x														x	x							
Polytmus guainumbi	LC	LC	LC							x	x		x			x			x							
Polytmus theresiae	LC	LC	LC	x						x								x	x							
Chrysolampis mosquitus	LC	LC	LC		x		x			x					x	x			x	x						
Anthracothorax prevostii	LC	LC	LC				x									x						x				
Anthracothorax nigricollis	LC	LC	LC	x	x		x			x			x		x	x		x	x	x						
Heliangelus mavors	LC	LC	LC		x	x									x											
Heliangelus amethysticollis	LC	LC	LC		x	x		x							x											
Heliangelus strophianus	LC	LC	LC		x	x									x											
Heliangelus exortis	LC	LC	LC		x	x									x											

Family and species	National category 2002	National category 2016	Global category 2018	ECOSYSTEMS L. rain-forest	Sub-A. forest	High A. forest	L. dry forest	Par-amo	Man-grove	Sav-anna	Fwt.	C.wt	REGIONS Pacific	P. Ocean	Andes	Caribbean	C. Sea	Amazon	Orinoquia	SNSM	Darién highlands	Sa&Pr.	GROUPS CONSERVATION CONCERN Game-birds	T. forest insectiv.	Large Frugiv.	Forest Raptors
Discosura conversii	LC	LC	LC	x	x								x		x						x					
Discosura popelairii	LC	LC	NT	x	x										x											
Discosura langsdorffi	LC	LC	LC	x														x								
Discosura longicaudus	LC	LC	LC	x														x								
Lophornis delattrei	LC	LC	LC	x	x								x		x											
Lophornis stictolophus	LC	LC	LC	x	x										x											
Lophornis chalybeus	LC	LC	NT	x														x	x							
Phlogophilus hemileucurus	NT	NT	VU		x										x											
Adelomyia melanogenys	LC	LC	LC	x	x	x									x											
Anthocephala floriceps	VU	VU	VU		x															x						
Anthocephala berlepschi	VU	VU	N/A*		x										x											
Aglaiocercus kingii	LC	LC	LC		x	x									x											
Aglaiocercus coelestis	LC	LC	LC	x	x										x											
Oreotrochilus chimborazo	LC	LC	LC					x							x											
Opisthoprora euryptera	LC	LC	LC			x									x											
Lesbia victoriae	LC	LC	LC			x		x							x											
Lesbia nuna	LC	LC	LC			x		x							x											
Ramphomicron dorsale	LC	LC	EN			x		x												x						
Ramphomicron	LC	LC	LC			x		x							x											
Chalcostigma stanleyi	LC	LC	LC					x							x											
Chalcostigma heteropogon	LC	LC	LC			x		x							x											
Chalcostigma herrani	LC	LC	LC			x		x							x											
Oxypogon stubelii	EN	EN	VU					x							x											
Oxypogon cyanolaemus	EN	EN	CR					x												x						
Oxypogon guerinii	LC	LC	LC					x							x											
Metallura tyrianthina	LC	LC	LC		x	x		x							x					x						
Metallura iracunda	VU	VU	EN			x		x							x											
Metallura williami	LC	LC	LC			x		x							x											
Haplophaedia aureliae	LC	LC	LC		x	x									x											
Haplophaedia lugens	NT	NT	NT		x										x											
Eriocnemis isabellae	CR	CR	CR			x									x											
Eriocnemis vestita	LC	LC	LC		x	x		x							x											
Eriocnemis derbyi	LC	LC	NT			x									x											
Eriocnemis godini	CR	CR	CR		x										x											
Eriocnemis cupreoventris	LC	LC	NT		x	x		x							x											
Eriocnemis luciani	LC	LC	LC			x		x							x											
Eriocnemis mosquera	LC	LC	LC			x		x							x											
Eriocnemis mirabilis	EN	EN	EN		x	x									x											
Eriocnemis aline	LC	LC	LC		x	x									x											
Aglaeactis cupripennis	LC	LC	LC			x		x							x											
Coeligena coeligena	LC	LC	LC		x	x									x											
Coeligena wilsoni	LC	LC	LC	x	x										x											
Coeligena prunellei	EN	NT	VU		x	x									x											
Coeligena torquata	LC	LC	LC		x	x		x							x											
Coeligena phalerata	LC	LC	NT		x	x		x												x						
Coeligena orina	EN	EN	CR			x		x							x											
Coeligena lutetiae	LC	LC	LC			x		x							x											
Coeligena bonapartei	LC	LC	LC		x	x		x							x											
Coeligena helianthea	LC	LC	LC		x	x		x							x											
Lafresnaya lafresnayi	LC	LC	LC		x	x		x							x					x						
Ensifera ensifera	LC	LC	LC			x		x							x											
Pterophanes cyanopterus	LC	LC	LC			x		x							x											
Boissonneaua flavescens	LC	LC	LC		x	x		x							x											
Boissonneaua matthewsii	LC	LC	LC		x										x											
Boissonneaua jardini	LC	LC	LC	x	x										x											
Ocreatus underwoodii	LC	LC	LC		x	x									x											
Urochroa bougueri	LC	LC	LC		x										x											

Family and species	National category 2002	National category 2016	Global category 2018	ECOSYSTEMS									REGIONS										GROUPS CONSERVATION CONCERN			
				L. rain-forest	Sub-A. forest	High A. forest	L. dry forest	Par-amo	Man-grove	Sav-anna	Fwt.	C.wt	Pacific	P. Ocean	Andes	Caribbean	C. Sea	Amazon	Orinoquia	SNSM	Darién highlands	Sa&Pr.	Game-birds	T. forest insectiv.	Large Frugiv.	Forest Raptors
Urosticte benjamini	LC	LC	LC	x	x										x											
Urosticte ruficrissa	LC	LC	LC		x										x											
Heliodoxa gularis	NT	NT	VU	x														x								
Heliodoxa schreibersii	LC	LC	LC	x	x										x			x								
Heliodoxa aurescens	LC	LC	LC	x	x										x			x								
Heliodoxa rubinoides	LC	LC	LC		x	x									x											
Heliodoxa jacula	LC	LC	LC	x	x										x						x					
Heliodoxa imperatrix	LC	LC	LC	x	x										x											
Heliodoxa leadbeateri	LC	LC	LC	x	x										x											
Patagona gigas	LC	LC	LC		x	x		x							x											
Sternoclyta cyanopectus	LC	LC	LC		x										x											
Heliomaster longirostris	LC	LC	LC	x	x		x						x		x	x		x	x	x	x					
Heliomaster furcifer	LC	LC	LC	x														x								
Chaetocercus mulsant	LC	LC	LC		x	x									x											
Chaetocercus heliodor	LC	LC	LC	x	x	x									x											
Chaetocercus astreans	LC	LC	LC	x	x	x														x						
Chaetocercus jourdanii	LC	LC	LC		x										x											
Calliphlox amethystina	LC	LC	LC	x														x	x							
Calliphlox mitchellii	LC	LC	LC	x	x										x											
Chlorostilbon melanorhynchus	LC	LC	N/A*	x	x										x											
Chlorostilbon gibsoni	LC	LC	LC	x	x		x								x	x				x						
Chlorostilbon mellisugus	LC	LC	LC	x	x										x	x		x	x							
Chlorostilbon olivaresi	LC	LC	LC	x														x								
Chlorostilbon russatus	LC	LC	LC	x	x										x					x						
Chlorostilbon stenurus	LC	LC	LC		x										x											
Chlorostilbon poortmani	LC	LC	LC	x	x	x									x											
Chlorestes notata	LC	LC	LC	x						x								x	x							
Klais guimeti	LC	LC	LC	x	x										x						x					
Phaeochroa cuvierii	LC	LC	LC				x									x										
Campylopterus largipennis	LC	LC	LC	x	x										x			x	x							
Campylopterus falcatus	LC	LC	LC	x	x	x									x					x						
Campylopterus phainopeplus	VU	VU	EN		x	x		x												x						
Campylopterus villaviscensio	LC	LC	NT	x	x										x											
Chalybura buffonii	LC	LC	LC	x	x								x		x	x				x						
Chalybura urochrysia	LC	LC	LC	x									x			x					x					
Thalurania colombica	LC	LC	LC	x	x								x		x	x				x						
Thalurania furcata	LC	LC	LC	x	x										x			x	x							
Taphrospilus hypostictus	LC	LC	LC	x	x										x											
Leucippus fallax	LC	LC	LC				x									x										
Leucippus chlorocercus	LC	LC	LC	x														x								
Amazilia tzacatl	LC	LC	LC	x	x		x						x		x	x				x	x					
Amazilia castaneiventris	EN	EN	EN		x										x											
Amazilia versicolor	LC	LC	LC	x						x								x	x							
Amazilia franciae	LC	LC	LC	x	x										x											
Amazilia fimbriata	LC	LC	LC	x	x										x	x		x	x							
Amazilia amabilis	LC	LC	LC	x	x		x						x		x	x										
Amazilia rosenbergi	LC	LC	LC	x									x													
Amazilia saucerottei	LC	LC	LC	x	x		x								x	x				x						
Amazilia cyanifrons	LC	LC	LC	x	x										x	x										
Amazilia edward	LC	LC	LC	x									x													
Amazilia viridigaster	LC	LC	LC	x	x										x											
Chrysuronia oenone	LC	LC	LC	x	x										x			x								
Goethalsia bella	VU	VU	NT		x																x					
Goldmania violiceps	LC	LC	NT	x	x																x					
Lepidopyga coeruleogularis	LC	LC	LC	x			x		x				x				x									
Lepidopyga lilliae	EN	EN	CR						x								x									
Lepidopyga goudoti	LC	LC	LC	x	x		x								x	x				x						

				ECOSYSTEMS									REGIONS										GROUPS CONSERVATION CONCERN			
**Family and species**	**National category 2002**	**National category 2016**	**Global category 2018**	**L. rain-forest**	**Sub-A. forest**	**High A. forest**	**L. dry forest**	**Par-amo**	**Man-grove**	**Sav-anna**	**Fwt.**	**C.wt**	**Pacific**	**P. Ocean**	**Andes**	**Caribbean**	**C. Sea**	**Amazon**	**Orinoquia**	**SNSM**	**Darién highlands**	**Sa&Pr.**	**Game-birds**	**T. forest insectiv.**	**Large Frugiv.**	**Forest Raptors**
Damophila julie	LC	LC	LC	x	x								x		x	x										
Hylocharis eliciae	LC	LC	LC	x									x													
Hylocharis sapphirina	LC	LC	LC	x														x	x							
Hylocharis cyanus	LC	LC	LC	x												x		x	x	x						
Hylocharis humboldtii	LC	LC	LC	x					x					x												
Hylocharis grayi	LC	LC	LC	x	x		x								x	x										
**Opisthocomidae**																										
Opisthocomus hoazin	LC	LC	LC	x														x	x							
**Aramidae**																										
Aramus guarauna	LC	LC	LC								x		x			x		x	x							
**Psophiidae**																										
Psophia crepitans	LC	LC	NT	x														x	x						x	
**Rallidae**																										
Coturnicops notatus	DD	DD	LC							x	x								x							
Micropygia schomburgkii	LC	LC	LC							x									x							
Rallus longirostris	LC	LC	LC						x			x		x			x									
Rallus limicola	LC	LC	LC								x				x											
Rallus semiplumbeus	EN	EN	EN								x				x											
Aramides wolfi	LC	LC	VU						x		x			x												
Aramides cajaneus	LC	LC	LC	x	x				x		x		x	x	x	x	x	x	x	x						
Aramides axillaris	LC	LC	LC	x					x					x			x									
Amaurolimnas concolor	LC	LC	LC	x									x													
Anurolimnas castaneiceps	LC	LC	LC	x														x								
Anurolimnas viridis	LC	LC	LC	x	x					x	x				x	x		x	x							
Anurolimnas fasciatus	LC	LC	LC								x							x								
Laterallus melanophaius	LC	LC	LC								x							x								
Laterallus albigularis	LC	LC	LC							x	x		x		x	x				x						
Laterallus exilis	LC	LC	LC								x		x		x	x		x								
Porzana flaviventer	LC	LC	LC								x					x			x							
Porzana carolina	LC	LC	LC								x		x		x	x			x	x		x				
Porphyriops melanops	EN	EN	LC								x				x											
Mustelirallus albicollis	LC	LC	LC								x					x			x							
Mustelirallus colombianus	DD	DD	DD							x	x		x		x	x					x					
Mustelirallus erythrops	LC	LC	LC								x				x				x							
Pardirallus maculatus	LC	LC	LC								x				x	x										
Pardirallus nigricans	LC	LC	LC								x				x	x										
Gallinula galeata	LC	LC	LC								x		x		x	x										
Porphyrio martinica	LC	LC	LC								x		x		x	x		x	x		x	x				
Porphyrio flavirostris	LC	LC	LC								x							x	x							
Fulica americana	LC	LC	LC								x				x	x										
Fulica ardesiaca	LC	LC	LC								x				x											
**Heliornithidae**																										
Heliornis fulica	LC	LC	LC								x		x		x	x		x	x							
**Charadriidae**																										
Pluvialis squatarola	LC	LC	LC									x		x			x					x				
Vanellus cayanus	LC	LC	LC								x							x	x							
Vanellus chilensis	LC	LC	LC							x	x		x		x	x		x	x	x						
Vanellus resplendens	LC	LC	LC					x							x											
Charadrius semipalmatus	LC	LC	LC						x			x		x			x					x				
Charadrius wilsonia	LC	LC	LC									x		x			x									
Charadrius collaris	LC	LC	LC								x	x	x	x	x	x	x	x	x	x						
**Haematopodidae**																										
Haematopus palliatus	LC	LC	LC									x		x			x									
**Recurvirostridae**																										
Himantopus mexicanus	LC	LC	N/A*								x	x	x	x	x	x	x		x							
**Burhinidae**																										
Burhinus bistriatus	LC	LC	LC							x						x			x							

Family and species	National category 2002	National category 2016	Global category 2018	ECOSYSTEMS									REGIONS										GROUPS CONSERVATION CONCERN			
				L. rain-forest	Sub-A. forest	High A. forest	L. dry forest	Par-amo	Man-grove	Sav-anna	Fwt.	C.wt	Pacific	P. Ocean	Andes	Caribbean	C. Sea	Amazon	Orinoquia	SNSM	Darién highlands	Sa&Pr.	Game-birds	T. forest insectiv.	Large Frugiv.	Forest Raptors
**Scolopacidae**																										
Numenius phaeopus	LC	LC	LC									x		x			x					x				
Arenaria interpres	LC	LC	LC									x		x			x					x				
Calidris canutus	LC	LC	NT									x		x			x									
Calidris virgata	LC	LC	LC									x		x												
Calidris himantopus	LC	LC	LC									x		x			x					x				
Calidris alba	LC	LC	LC									x		x			x					x				
Calidris minutilla	LC	LC	LC								x	x	x	x	x	x	x	x	x			x				
Calidris melanotos	LC	LC	LC								x	x	x	x	x	x	x	x	x			x				
Calidris pusilla	LC	LC	NT									x		x			x					x				
Calidris mauri	LC	LC	LC									x		x			x									
Limnodromus griseus	LC	LC	LC								x	x		x			x					x				
Gallinago imperialis	DD	DD	NT			x		x			x				x											
Gallinago jamesoni	LC	LC	LC					x			x				x					x						
Gallinago nobilis	LC	LC	NT					x			x				x											
Gallinago undulata	LC	LC	LC							x	x							x	x							
Gallinago delicata	LC	LC	LC					x			x		x		x	x		x	x			x				
Gallinago paraguaiae	LC	LC	LC							x	x							x	x							
Actitis macularius	LC	LC	LC								x	x	x	x	x	x	x	x	x	x		x				
Tringa solitaria	LC	LC	LC								x	x	x	x	x	x	x	x	x	x		x				
Tringa melanoleuca	LC	LC	LC								x	x	x	x	x	x	x	x	x	x						
Tringa semipalmata	LC	LC	LC									x		x			x					x				
Tringa flavipes	LC	LC	LC								x	x	x	x	x	x	x	x	x	x		x				
**Jacanidae**																										
Jacana jacana	LC	LC	LC								x		x		x	x		x	x		x					
**Stercorariidae**																										
Stercorarius pomarinus	LC	LC	LC									x		x			x					x				
**Laridae**																										
Creagrus furcatus	EN	EN	LC									x		x												
Chroicocephalus serranus	LC	LC	LC								x				x											
Leucophaeus atricilla	LC	LC	LC								x	x	x	x	x	x	x									
Anous stolidus	LC	LC	LC									x		x			x									
Anous minutus	LC	LC	LC									x		x												
Gygis alba	LC	LC	LC									x		x												
Onychoprion anaethetus	LC	LC	LC									x		x			x									
Sternula antillarum	LC	LC	LC								x	x					x									
Sternula superciliaris	LC	LC	LC								x					x		x	x							
Phaetusa simplex	LC	LC	LC								x				x	x		x	x							
Gelochelidon nilotica	LC	LC	LC									x		x			x									
Hydroprogne caspia	LC	LC	LC								x	x		x			x									
Chlidonias niger	LC	LC	LC									x		x			x					x				
Thalasseus elegans	LC	LC	NT									x		x			x									
Thalasseus sandvicensis	LC	LC	LC									x		x			x									
Thalasseus maximus	LC	LC	LC									x		x			x					x				
**Rynchopidae**																										
Rynchops niger	LC	LC	LC								x		x			x		x	x							
**Eurypygidae**																										
Eurypyga helias	LC	LC	LC	x							x		x			x		x	x							
**Phaethontidae**																										
Phaethon aethereus	LC	LC	LC									x		x			x									
**Procellariidae**																										
Puffinus lherminieri	LC	LC	LC									x		x			x					x				
**Hydrobatidae**																										
Oceanodroma melania	LC	LC	LC									x		x												
**Ciconiidae**																										
Ciconia maguari	LC	LC	LC							x	x		x						x							
Jabiru mycteria	LC	LC	LC							x	x					x		x	x							

				ECOSYSTEMS									REGIONS										GROUPS CONSERVATION CONCERN			
Family and species	National category 2002	National category 2016	Global category 2018	L. rain-forest	Sub-A. forest	High A. forest	L. dry forest	Par-amo	Man-grove	Sav-anna	Fwt.	C.wt	Pacific	P. Ocean	Andes	Caribbean	C. Sea	Amazon	Orinoquia	SNSM	Darién highlands	Sa&Pr.	Game-birds	T. forest insectiv.	Large Frugiv.	Forest Raptors
Mycteria americana	LC	LC	LC						x		x				x	x	x	x	x							
**Fregatidae**																										
Fregata magnificens	LC	LC	LC									x		x			x					x				
**Sulidae**																										
Sula nebouxii	LC	LC	LC									x		x			x									
Sula dactylatra	LC	LC	LC									x		x			x									
Sula granti	VU	VU	LC									x		x												
Sula leucogaster	EN	EN	LC									x		x		x	x					x				
**Phalacrocoracidae**																										
Phalacrocorax brasilianus	LC	LC	LC						x		x	x	x	x	x	x	x	x	x	x	x					
**Anhingidae**																										
Anhinga anhinga	LC	LC	LC								x		x		x	x		x	x							
**Pelecanidae**																										
Pelecanus occidentalis	LC	LC	LC									x		x			x					x				
**Ardeidae**																										
Tigrisoma lineatum	LC	LC	LC								x		x			x		x	x							
Tigrisoma fasciatum	LC	LC	LC								x		x		x	x				x						
Tigrisoma mexicanum	LC	LC	LC						x		x			x			x									
Agamia agami	LC	LC	VU						x		x		x	x	x	x	x	x	x							
Cochlearius cochlearius	LC	LC	LC						x		x			x	x	x	x	x	x		x					
Zebrilus undulatus	LC	LC	NT								x							x	x							
Botaurus pinnatus	LC	LC	LC								x				x	x			x							
Ixobrychus exilis	LC	LC	LC								x				x	x		x	x							
Ixobrychus involucris	LC	LC	LC								x					x										
Nycticorax nycticorax	LC	LC	LC						x		x		x	x	x	x	x	x	x							
Nyctanassa violacea	LC	LC	LC						x			x		x		x	x		x			x				
Butorides virescens	LC	LC	N/A*						x		x	x	x	x	x	x	x		x			x				
Butorides striata	LC	LC	LC						x		x	x	x	x	x	x	x	x	x		x	x				
Bubulcus ibis	LC	LC	LC							x			x		x	x		x	x	x	x	x				
Ardea herodias	LC	LC	LC						x		x	x	x	x	x	x	x		x			x				
Ardea cocoi	LC	LC	LC						x		x	x	x	x	x	x	x	x	x							
Ardea alba	LC	LC	LC						x		x	x	x	x	x	x	x	x	x			x				
Syrigma sibilatrix	LC	LC	LC							x	x					x		x	x							
Pilherodius pileatus	LC	LC	LC								x					x		x	x	x						
Egretta tricolor	LC	LC	LC									x		x			x					x				
Egretta rufescens	VU	VU	NT									x				x	x									
Egretta thula	LC	LC	LC						x		x	x	x	x	x	x	x	x	x	x		x				
Egretta caerulea	LC	LC	LC						x		x	x	x	x	x	x	x	x	x			x				
**Threskiornithidae**																										
Eudocimus albus	LC	LC	LC						x		x	x		x		x	x		x							
Eudocimus ruber	LC	LC	LC								x	x				x	x	x	x							
Plegadis falcinellus	LC	LC	LC								x		x			x			x							
Cercibis oxycerca	LC	LC	LC								x								x							
Mesembrinibis cayennensis	LC	LC	LC								x		x			x		x	x							
Phimosus infuscatus	LC	LC	LC							x	x		x		x	x		x	x							
Theristicus caudatus	LC	LC	LC							x	x				x	x			x							
Platalea ajaja	LC	LC	LC						x		x	x				x	x	x	x							
**Cathartidae**																										
Cathartes aura	LC	LC	LC	x	x	x	x			x			x		x	x		x	x	x	x					
Cathartes burrovianus	LC	LC	LC							x	x				x	x		x	x	x						
Cathartes melambrotus	LC	LC	LC	x	x					x								x	x							
Coragyps atratus	LC	LC	LC							x	x		x		x	x		x	x	x	x					
Sarcoramphus papa	LC	LC	LC	x	x		x						x		x	x		x	x	x						
Vultur gryphus	CR	CR	NT					x							x					x						
**Pandionidae**																										
Pandion haliaetus	LC	LC	LC						x		x	x	x	x	x	x	x	x	x	x		x				
**Accipitridae**																										

Family and species	National category 2002	National category 2016	Global category 2018	ECOSYSTEMS L. rain-forest	Sub-A. forest	High A. forest	L. dry forest	Par-amo	Man-grove	Sav-anna	Fwt.	C.wt	REGIONS Pacific	P. Ocean	Andes	Caribbean	C. Sea	Amazon	Orinoquia	SNSM	Darién highlands	Sa&Pr.	GROUPS CONSERVATION CONCERN Game-birds	T. forest insectiv.	Large Frugiv.	Forest Raptors
Elanus leucurus	LC	LC	LC							x					x	x			x							
Gampsonyx swainsonii	LC	LC	LC	x	x		x			x					x	x		x	x	x						
Chondrohierax uncinatus	LC	LC	LC	x	x	x					x				x	x		x	x	x						x
Leptodon cayanensis	LC	LC	LC	x			x						x			x		x	x	x	x					x
Elanoides forficatus	LC	LC	LC	x	x	x			x				x	x	x	x	x	x	x	x	x					x
Morphnus guianensis	NT	NT	NT	x									x		x			x	x		x					x
Harpia harpyja	NT	NT	NT	x									x		x			x	x							x
Spizaetus tyrannus	LC	LC	LC	x	x								x		x	x		x	x	x						x
Spizaetus melanoleucus	LC	LC	LC	x	x								x		x	x		x	x							x
Spizaetus ornatus	LC	LC	NT	x	x								x		x	x		x	x	x	x					x
Spizaetus isidori	EN	EN	EN		x	x									x					x						x
Busarellus nigricollis	LC	LC	LC	x					x		x		x	x		x	x	x	x							
Rostrhamus sociabilis	LC	LC	LC								x		x		x	x		x	x							
Helicolestes hamatus	LC	LC	LC								x		x					x	x							x
Harpagus bidentatus	LC	LC	LC	x	x								x		x	x		x	x	x						x
Ictinia plumbea	LC	LC	LC	x	x	x							x		x	x		x	x	x						x
Circus cyaneus	LC	LC	LC							x	x		x		x	x										
Circus cinereus	EN	EN	LC							x	x				x											
Circus buffoni	LC	LC	LC							x	x					x			x							
Accipiter poliogaster	LC	LC	NT	x												x		x	x							x
Accipiter superciliosus	LC	LC	LC	x	x								x		x	x		x	x	x	x					x
Accipiter collaris	NT	NT	NT	x	x										x	x				x						x
Accipiter striatus	LC	LC	LC		x	x									x					x						x
Accipiter cooperii	LC	LC	LC		x	x							x		x	x										
Accipiter bicolor	LC	LC	LC	x	x		x						x		x	x		x	x	x						x
Geranospiza caerulescens	LC	LC	LC	x			x						x			x		x	x	x						
Cryptoleucopteryx plumbea	NT	NT	VU	x									x								x					x
Buteogallus schistaceus	LC	LC	LC	x							x							x	x							
Buteogallus anthracinus	LC	LC	LC	x					x		x	x		x		x	x			x						
Buteogallus meridionalis	LC	LC	LC							x					x	x			x							
Buteogallus urubitinga	LC	LC	LC	x					x		x		x	x		x	x	x	x	x	x					
Buteogallus solitarius	CR	CR	NT	x	x										x					x						x
Morphnarchus princeps	LC	LC	LC	x	x										x											x
Rupornis magnirostris	LC	LC	LC	x	x		x			x			x		x	x		x	x	x						
Parabuteo unicinctus	LC	LC	LC		x		x								x	x										
Parabuteo leucorrhous	LC	LC	LC		x	x									x					x						x
Geranoaetus albicaudatus	LC	LC	LC							x					x	x			x	x						
Geranoaetus polyosoma	LC	LC	LC			x		x							x											
Geranoaetus melanoleucus	LC	LC	LC		x	x		x							x					x						
Pseudastur albicollis	LC	LC	LC	x	x								x		x	x		x	x							
Leucopternis semiplumbeus	LC	LC	LC	x									x			x										x
Leucopternis melanops	LC	LC	LC	x														x	x							x
Buteo nitidus	LC	LC	LC	x			x								x	x		x	x	x						
Buteo platypterus	LC	LC	LC	x	x	x	x	x		x			x		x	x		x	x	x	x					
Buteo albigula	LC	LC	LC		x	x									x											
Buteo brachyurus	LC	LC	LC	x	x	x	x						x		x	x		x	x	x						
Buteo albonotatus	LC	LC	LC	x			x			x						x		x	x	x						x
**Tytonidae (nocturnal raptors)**																										
Tyto alba	LC	LC	LC							x			x		x	x		x	x			x				
**Strigidae (nocturnal raptors)**																										
Megascops choliba	LC	LC	LC	x	x	x	x			x			x		x	x		x	x	x						
Megascops clarkii	LC	LC	LC		x																x					
Megascops colombianus	LC	LC	NT		x										x											
Megascops ingens	LC	LC	LC		x										x											
Megascops petersoni	LC	LC	LC		x										x											
Megascops watsonii	LC	LC	LC	x														x	x							
Megascops guatemalae	LC	LC	LC	x	x								x		x	x										

Family and species	National category 2002	National category 2016	Global category 2018	ECOSYSTEMS									REGIONS										GROUPS CONSERVATION CONCERN			
				L. rain-forest	Sub-A. forest	High A. forest	L. dry forest	Par-amo	Man-grove	Sav-anna	Fwt.	C.wt	Pacific	P. Ocean	Andes	Caribbean	C. Sea	Amazon	Orinoquia	SNSM	Darién highlands	Sa&Pr.	Game-birds	T. forest insectiv.	Large Frugiv.	Forest Raptors
Megascops albogularis	LC	LC	LC		x	x									x											
Lophostrix cristata	LC	LC	LC	x									x			x		x								
Pulsatrix perspicillata	LC	LC	LC	x	x								x		x	x		x	x	x	x					
Pulsatrix melanota	LC	LC	LC	x	x										x											
Bubo virginianus	LC	LC	LC					x		x					x	x			x							
Ciccaba virgata	LC	LC	LC	x	x								x		x	x		x		x	x					
Ciccaba nigrolineata	LC	LC	LC	x	x								x		x	x				x						
Ciccaba huhula	LC	LC	LC	x														x	x							
Ciccaba albitarsis	LC	LC	LC		x	x									x											
Glaucidium nubicola	VU	VU	VU		x										x											
Glaucidium jardinii	LC	LC	LC		x	x									x											
Glaucidium parkeri	LC	LC	LC		x										x											
Glaucidium griseiceps	LC	LC	LC	x	x								x		x	x										
Glaucidium brasilianum	LC	LC	LC	x			x									x		x	x	x						
Athene cunicularia	LC	LC	LC				x			x						x			x							
Aegolius harrisii	LC	LC	LC		x	x									x											
Asio clamator	LC	LC	LC							x					x	x		x								
Asio stygius	LC	LC	LC		x	x									x					x						
Asio flammeus	LC	LC	LC					x		x					x				x							
**Trogonidae**																										
Pharomachrus pavoninus	LC	LC	LC	x														x							x	
Pharomachrus auriceps	LC	LC	LC	x	x	x									x										x	
Pharomachrus fulgidus	LC	LC	LC		x	x														x					x	
Pharomachrus antisianus	LC	LC	LC		x	x									x										x	
Trogon massena	LC	LC	LC	x									x												x	
Trogon comptus	LC	LC	LC	x	x								x		x	x									x	
Trogon melanurus	LC	LC	LC	x									x		x	x		x	x						x	
Trogon chionurus	LC	LC	LC	x	x								x		x	x										
Trogon viridis	LC	LC	LC	x	x										x			x	x							
Trogon caligatus	LC	LC	LC	x	x		x						x		x	x				x						
Trogon ramonianus	LC	LC	LC	x														x	x							
Trogon curucui	LC	LC	LC	x														x	x							
Trogon rufus	LC	LC	LC	x	x								x		x	x		x	x							
Trogon collaris	LC	LC	LC	x	x	x									x			x	x		x					
Trogon personatus	LC	LC	LC		x	x									x					x						
**Alcedinidae**																										
Megaceryle torquata	LC	LC	LC						x		x		x	x	x	x	x	x	x	x						
Megaceryle alcyon	LC	LC	LC						x		x			x			x					x				
Chloroceryle amazona	LC	LC	LC								x		x		x	x		x	x	x						
Chloroceryle americana	LC	LC	LC						x		x		x	x	x	x	x	x	x	x	x					
Chloroceryle inda	LC	LC	LC						x		x		x	x		x	x	x	x							
Chloroceryle aenea	LC	LC	LC						x		x		x	x		x	x	x	x							
**Momotidae**																										
Hylomanes momotula	LC	LC	LC	x	x											x					x					
Electron platyrhynchum	LC	LC	LC	x									x			x		x								
Baryphthengus martii	LC	LC	LC	x	x								x		x	x		x							x	
Momotus subrufescens	LC	LC	LC	x	x								x		x	x				x					x	
Momotus momota	LC	LC	LC	x	x		x											x	x						x	
Momotus aequatorialis	LC	LC	LC		x	x									x										x	
**Galbulidae**																										
Galbalcyrhynchus leucotis	LC	LC	LC	x														x								
Brachygalba lugubris	LC	LC	LC							x								x	x							
Brachygalba goeringi	LC	LC	LC				x												x							
Brachygalba salmoni	LC	LC	LC	x									x			x										
Galbula albirostris	LC	LC	LC	x														x	x							
Galbula ruficauda	LC	LC	LC	x	x		x						x		x	x			x	x						
Galbula galbula	LC	LC	LC	x			x												x							

Family and species	National category 2002	National category 2016	Global category 2018	ECOSYSTEMS L. rain-forest	Sub-A. forest	High A. forest	L. dry forest	Par-amo	Man-grove	Sav-anna	Fwt.	C.wt	REGIONS Pacific	P. Ocean	Andes	Caribbean	C. Sea	Amazon	Orinoquia	SNSM	Darién highlands	Sa&Pr.	GROUPS CONSERVATION CONCERN Game-birds	T. forest insectiv.	Large Frugiv.	Forest Raptors
Galbula tombacea	LC	LC	LC	x														x								
Galbula pastazae	VU	VU	VU		x										x											
Galbula chalcothorax	LC	LC	LC	x														x								
Galbula leucogastra	LC	LC	LC	x														x								
Galbula dea	LC	LC	LC	x			x											x	x							
Jacamerops aureus	LC	LC	LC	x									x			x		x	x							
**Bucconidae**																										
Notharchus hyperrhynchus	LC	LC	LC	x	x								x		x	x		x								
Notharchus pectoralis	LC	LC	LC	x									x			x										
Notharchus ordii	LC	LC	LC	x														x	x							
Notharchus tectus	LC	LC	LC	x					x				x	x		x	x	x								
Bucco macrodactylus	LC	LC	LC	x														x	x							
Bucco tamatia	LC	LC	LC	x														x	x							
Bucco noanamae	NT	NT	NT	x									x													
Bucco capensis	LC	LC	LC	x														x	x							
Nystalus radiatus	LC	LC	LC	x	x								x		x	x										
Hypnelus ruficollis	LC	LC	LC		x		x								x	x			x	x						
Malacoptila fusca	LC	LC	LC	x														x	x							
Malacoptila panamensis	LC	LC	LC	x	x								x		x	x										
Malacoptila fulvogularis	LC	LC	LC	x	x										x											
Malacoptila mystacalis	LC	LC	LC	x	x										x					x						
Micromonacha lanceolata	LC	LC	LC	x	x								x		x			x								
Nonnula rubecula	LC	LC	LC	x														x								
Nonnula brunnea	LC	LC	LC	x														x	x							
Nonnula frontalis	LC	LC	LC	x									x			x										
Hapaloptila castanea	LC	LC	LC	x	x										x											
Monasa nigrifrons	LC	LC	LC	x														x	x							
Monasa morphoeus	LC	LC	LC	x	x								x		x	x		x								
Monasa flavirostris	LC	LC	LC	x														x								
Chelidoptera tenebrosa	LC	LC	LC	x														x	x							
**Capitonidae**																										
Capito aurovirens	LC	LC	LC	x														x								
Capito maculicoronatus	LC	LC	LC	x									x			x										
Capito squamatus	NT	VU	NT	x									x													
Capito hypoleucus	EN	EN	VU	x	x										x	x										
Capito quinticolor	NT	NT	VU	x									x													
Capito auratus	LC	LC	LC	x	x										x			x								
Eubucco richardsoni	LC	LC	LC	x														x								
Eubucco bourcierii	LC	LC	LC	x	x	x									x						x					
**Semnornithidae**																										
Semnornis ramphastinus	NT	NT	NT		x										x											
**Ramphastidae**																										
Ramphastos ambiguus	LC	LC	NT	x	x								x		x	x									x	
Ramphastos tucanus	LC	LC	VU	x	x													x	x						x	
Ramphastos sulfuratus	LC	LC	LC	x	x		x								x	x				x					x	
Ramphastos brevis	LC	LC	LC	x	x								x		x										x	
Ramphastos vitellinus	LC	LC	VU	x	x										x	x		x	x						x	
Aulacorhynchus prasinus	LC	LC	LC		x	x									x					x	x				x	
Aulacorhynchus sulcatus	LC	LC	LC	x	x										x					x					x	
Aulacorhynchus derbianus	LC	LC	LC	x	x										x										x	
Aulacorhynchus haematopygus	LC	LC	LC	x	x										x										x	
Andigena hypoglauca	VU	VU	NT			x									x										x	
Andigena laminirostris	VU	EN	NT		x	x									x										x	
Andigena nigrirostris	NT	NT	LC		x	x									x										x	
Selenidera spectabilis	LC	LC	LC	x	x								x		x	x					x				x	
Selenidera reinwardtii	LC	LC	LC	x	x										x			x							x	
Selenidera nattereri	LC	LC	LC	x														x							x	

Family and species	National category 2002	National category 2016	Global category 2018	ECOSYSTEMS L. rain-forest	Sub-A. forest	High A. forest	L. dry forest	Par-amo	Man-grove	Sav-anna	Fwt.	C.wt	REGIONS Pacific	P. Ocean	Andes	Caribbean	C. Sea	Amazon	Orinoquia	SNSM	Darién highlands	Sa&Pr.	GROUPS CONSERVATION CONCERN Game-birds	T. forest insectiv.	Large Frugiv.	Forest Raptors
Pteroglossus inscriptus	LC	LC	LC	x														x	x						x	
Pteroglossus torquatus	LC	LC	LC	x	x		x						x		x	x				x					x	
Pteroglossus castanotis	LC	LC	LC	x	x					x					x			x	x						x	
Pteroglossus pluricinctus	LC	LC	LC	x	x										x			x	x						x	
Pteroglossus azara	LC	LC	LC	x	x										x			x	x						x	
**Picidae**																										
Picumnus pumilus	LC	LC	LC	x						x								x	x							
Picumnus lafresnayi	LC	LC	LC	x	x										x			x	x							
Picumnus exilis	LC	LC	LC	x														x	x							
Picumnus squamulatus	LC	LC	LC		x		x								x	x		x	x	x						
Picumnus rufiventris	LC	LC	LC	x	x										x			x								
Picumnus castelnau	LC	LC	LC	x														x								
Picumnus olivaceus	LC	LC	LC	x	x		x						x		x	x										
Picumnus granadensis	LC	LC	LC	x	x										x											
Picumnus cinnamomeus	LC	LC	LC				x		x							x	x									
Melanerpes formicivorus	LC	LC	LC		x	x									x											
Melanerpes cruentatus	LC	LC	LC	x	x					x					x			x	x							
Melanerpes pulcher	LC	LC	LC	x	x										x	x										
Melanerpes pucherani	LC	LC	LC	x									x								x					
Melanerpes rubricapillus	LC	LC	LC		x		x		x				x	x	x	x	x		x	x						
Picoides fumigatus	LC	LC	LC		x	x									x					x	x					
Veniliornis kirkii	LC	LC	LC	x	x				x				x	x	x	x	x									
Veniliornis passerinus	LC	LC	LC	x	x		x			x					x			x	x							
Veniliornis callonotus	EN	EN	LC				x						x													
Veniliornis dignus	LC	LC	LC		x	x									x											
Veniliornis nigriceps	LC	LC	LC		x	x									x											
Veniliornis affinis	LC	LC	LC	x	x										x			x	x							
Veniliornis chocoensis	LC	LC	NT	x									x													
Piculus leucolaemus	LC	LC	LC	x	x								x		x											
Piculus litae	LC	LC	LC	x	x								x		x	x										
Piculus flavigula	LC	LC	LC	x														x	x							
Piculus chrysochloros	LC	LC	LC	x	x										x	x		x	x							
Colaptes rubiginosus	LC	LC	LC	x	x	x									x					x						
Colaptes rivolii	LC	LC	LC		x	x		x							x											
Colaptes punctigula	LC	LC	LC	x	x		x		x				x	x	x	x	x	x	x							
Celeus loricatus	LC	LC	LC	x	x								x		x	x										
Celeus grammicus	LC	LC	LC	x														x	x							
Celeus elegans	LC	LC	LC	x														x	x						x	
Celeus flavus	LC	LC	LC	x														x	x						x	
Celeus torquatus	LC	LC	NT	x														x	x							
Dryocopus lineatus	LC	LC	LC	x	x								x		x	x		x	x	x						
Campephilus pollens	LC	LC	LC	x	x	x									x											
Campephilus haematogaster	LC	LC	LC	x	x								x		x	x					x					
Campephilus rubricollis	LC	LC	LC	x														x	x							
Campephilus melanoleucos	LC	LC	LC	x	x								x		x	x		x	x	x	x					
Campephilus gayaquilensis	NT	EN	NT	x					x				x	x												
**Falconidae**																										
Herpetotheres cachinnans	LC	LC	LC	x	x		x			x			x		x	x		x	x	x						
Micrastur ruficollis	LC	LC	LC	x	x								x		x	x		x	x	x	x					x
Micrastur plumbeus	NT	EN	VU	x									x													x
Micrastur gilvicollis	LC	LC	LC	x	x										x			x	x							x
Micrastur mirandollei	LC	LC	LC	x									x					x								x
Micrastur semitorquatus	LC	LC	LC	x	x								x		x	x		x	x	x						x
Caracara cheriway	LC	LC	LC	x	x	x	x			x			x		x	x			x	x						
Ibycter americanus	LC	LC	LC	x	x								x		x	x		x	x							x
Phalcoboenus carunculatus	LC	LC	LC			x		x							x											
Daptrius ater	LC	LC	LC	x														x	x							

Family and species	National category 2002	National category 2016	Global category 2018	ECOSYSTEMS									REGIONS										GROUPS CONSERVATION CONCERN			
				L. rain-forest	Sub-A. forest	High A. forest	L. dry forest	Par-amo	Man-grove	Sav-anna	Fwt.	C.wt	Pacific	P. Ocean	Andes	Caribbean	C. Sea	Amazon	Orinoquia	SNSM	Darién highlands	Sa&Pr.	Game-birds	T. forest insectiv.	Large Frugiv.	Forest Raptors
Milvago chimachima	LC	LC	LC	x	x					x			x		x	x		x	x	x						
Falco sparverius	LC	LC	LC							x			x		x	x			x	x		x				
Falco columbarius	LC	LC	LC							x			x		x	x			x	x		x				
Falco rufigularis	LC	LC	LC	x	x		x						x		x	x		x	x	x						
Falco deiroleucus	DD	DD	NT	x	x										x	x		x	x							x
Falco femoralis	LC	LC	LC	x	x					x					x	x			x	x						
Falco peregrinus	LC	LC	LC							x			x	x	x	x		x	x	x						
**Psittacidae (parrots)**																										
Touit batavicus	LC	LC	LC		x															x						
Touit huetii	LC	LC	VU	x														x	x							
Touit dilectissimus	LC	LC	LC	x	x								x		x						x					
Touit purpuratus	LC	LC	LC	x														x	x							
Touit stictopterus	VU	VU	VU		x										x											
Bolborhynchus lineola	LC	LC	LC		x	x									x					x						
Bolborhynchus ferrugineifrons	VU	VU	VU			x		x							x											
Brotogeris sanctithomae	LC	LC	LC	x														x								
Brotogeris versicolurus	LC	LC	LC	x														x								
Brotogeris jugularis	LC	LC	LC	x	x		x						x		x	x			x	x						
Brotogeris cyanoptera	LC	LC	LC	x						x								x	x							
Hapalopsittaca amazonina	VU	VU	VU		x	x									x										x	
Hapalopsittaca fuertesi	CR	CR	CR			x									x										x	
Pyrilia haematotis	LC	LC	LC	x	x								x		x						x				x	
Pyrilia pulchra	LC	LC	LC	x	x								x		x										x	
Pyrilia pyrilia	NT	NT	NT	x									x		x	x									x	
Pyrilia barrabandi	LC	LC	NT	x														x	x						x	
Pionus fuscus	EN	EN	LC		x										x										x	
Pionus sordidus	LC	LC	LC	x	x		x								x					x					x	
Pionus tumultuosus	LC	LC	LC		x	x									x										x	
Pionus menstruus	LC	LC	LC	x	x								x		x	x		x	x	x					x	
Pionus chalcopterus	LC	LC	LC	x	x										x										x	
Graydidascalus brachyurus	LC	LC	LC	x														x							x	
Amazona festiva	LC	LC	NT	x														x	x						x	
Amazona autumnalis	LC	LC	LC	x	x								x		x	x									x	
Amazona ochrocephala	LC	LC	LC	x	x		x			x			x		x	x		x	x						x	
Amazona farinosa	LC	LC	NT	x	x								x		x	x		x	x						x	
Amazona amazonica	LC	LC	LC	x	x		x		x						x	x	x	x	x	x					x	
Amazona mercenarius	LC	LC	LC		x	x									x					x					x	
Forpus passerinus	LC	LC	LC				x									x										
Forpus xanthopterygius	LC	LC	LC	x			x									x		x								
Forpus conspicillatus	LC	LC	LC		x		x			x					x	x			x							
Forpus modestus	LC	LC	LC	x														x	x							
Pionites melanocephalus	LC	LC	LC	x														x	x						x	
Pionites leucogaster	LC	LC	EN	x														x							x	
Deroptyus accipitrinus	LC	LC	LC	x						x								x	x						x	
Pyrrhura picta	LC	LC	LC	x	x										x											
Pyrrhura viridicata	EN	EN	EN		x	x														x						
Pyrrhura melanura	LC	LC	LC	x	x										x			x	x							
Pyrrhura calliptera	VU	VU	VU		x	x		x							x											
Eupsittula pertinax	LC	LC	LC				x		x	x						x	x		x	x						
Aratinga weddellii	LC	LC	LC	x														x							x	
Orthopsittaca manilatus	LC	LC	LC	x						x								x	x						x	
Ara ararauna	LC	LC	LC	x	x								x		x	x		x	x						x	
Ara militaris	VU	VU	VU	x	x		x								x	x				x					x	
Ara ambiguus	EN	EN	EN	x									x								x				x	
Ara macao	LC	LC	LC	x	x										x	x		x	x						x	
Ara chloropterus	LC	LC	LC	x						x			x			x		x	x		x				x	
Ara severus	LC	LC	LC	x	x					x			x		x	x		x	x						x	

Family and species	National category 2002	National category 2016	Global category 2018	ECOSYSTEMS									REGIONS										GROUPS CONSERVATION CONCERN			
				L. rain-forest	Sub-A. forest	High A. forest	L. dry forest	Par-amo	Man-grove	Sav-anna	Fwt.	C.wt	Pacific	P. Ocean	Andes	Caribbean	C. Sea	Amazon	Orinoquia	SNSM	Darién highlands	Sa&Pr.	Game-birds	T. forest insectiv.	Large Frugiv.	Forest Raptors
Leptosittaca branickii	VU	VU	VU		x	x									x										x	
Ognorhynchus icterotis	CR	EN	EN		x	x									x										x	
Thectocercus acuticaudatus	LC	LC	LC				x									x			x						x	
Psittacara wagleri	LC	LC	NT	x	x	x									x	x				x					x	
Psittacara leucophthalmus	LC	LC	LC	x														x	x						x	
**SUBOSCINES**																										
**Sapayoidae**																										
Sapayoa aenigma	LC	LC	LC	x									x													
**Thamnophilidae**																										
Euchrepomis callinota	LC	LC	LC	x	x										x						x					
Euchrepomis spodioptila	LC	LC	LC	x														x	x							
Cymbilaimus lineatus	LC	LC	LC	x	x								x		x	x		x	x							
Frederickena fulva	LC	LC	LC	x														x								
Taraba major	LC	LC	LC	x	x		x						x		x	x		x	x							
Sakesphorus canadensis	LC	LC	LC	x			x		x							x	x	x	x							
Thamnophilus doliatus	LC	LC	LC	x	x		x								x	x		x	x	x						
Thamnophilus multistriatus	LC	LC	LC		x										x											
Thamnophilus tenuepunctatus	LC	LC	VU		x										x											
Thamnophilus atrinucha	LC	LC	LC	x	x								x		x	x				x						
Thamnophilus schistaceus	LC	LC	LC	x														x								
Thamnophilus murinus	LC	LC	LC	x														x	x							
Thamnophilus nigriceps	LC	LC	LC		x		x								x	x										
Thamnophilus praecox	LC	LC	NT	x														x								
Thamnophilus cryptoleucus	LC	LC	NT	x														x								
Thamnophilus nigrocinereus	LC	LC	NT	x														x	x							
Thamnophilus punctatus	LC	LC	LC	x											x				x							
Thamnophilus unicolor	LC	LC	LC	x	x										x											
Thamnophilus aethiops	LC	LC	LC	x														x	x							
Thamnophilus melanonotus	LC	LC	LC				x									x				x						
Thamnophilus amazonicus	LC	LC	LC	x														x	x							
Megastictus margaritatus	LC	LC	LC	x														x	x							
Neoctantes niger	LC	LC	LC	x														x								
Clytoctantes alixii	VU	VU	EN	x	x										x	x										
Thamnistes anabatinus	LC	LC	LC	x	x								x		x											
Dysithamnus mentalis	LC	LC	LC	x	x										x					x	x					
Dysithamnus puncticeps	LC	LC	LC	x									x													
Dysithamnus occidentalis	VU	VU	VU		x										x											
Dysithamnus leucostictus	LC	LC	VU	x	x										x											
Thamnomanes ardesiacus	LC	LC	LC	x														x	x							
Thamnomanes caesius	LC	LC	LC	x														x	x							
Xenornis setifrons	VU	VU	VU	x																	x					
Isleria hauxwelli	LC	LC	LC	x														x	x							
Pygiptila stellaris	LC	LC	LC	x														x	x							
Epinecrophylla fulviventris	LC	LC	LC	x									x			x										
Epinecrophylla haematonota	LC	LC	LC	x	x													x	x							
Epinecrophylla spodionota	LC	LC	LC	x	x										x											
Epinecrophylla ornata	LC	LC	LC	x	x										x			x								
Epinecrophylla erythrura	LC	LC	LC	x														x								
Myrmotherula brachyura	LC	LC	LC	x														x	x							
Myrmotherula ignota	LC	LC	LC	x									x			x		x								
Myrmotherula ambigua	LC	LC	LC	x														x								
Myrmotherula multostriata	LC	LC	LC	x														x	x							
Myrmotherula pacifica	LC	LC	LC	x	x								x		x	x										
Myrmotherula cherriei	LC	LC	LC	x						x								x	x							
Myrmotherula longicauda	LC	LC	LC	x	x										x			x								
Myrmotherula axillaris	LC	LC	LC	x									x			x		x	x	x						
Myrmotherula schisticolor	LC	LC	LC		x										x					x	x					

Family and species	National category 2002	National category 2016	Global category 2018	ECOSYSTEMS									REGIONS										GROUPS CONSERVATION CONCERN			
				L. rain-forest	Sub-A. forest	High A. forest	L. dry forest	Par-amo	Man-grove	Sav-anna	Fwt.	C.wt	Pacific	P. Ocean	Andes	Caribbean	C. Sea	Amazon	Orinoquia	SNSM	Darién highlands	Sa&Pr.	Game-birds	T. forest insectiv.	Large Frugiv.	Forest Raptors
Myrmotherula sunensis	LC	LC	LC	x														x								
Myrmotherula longipennis	LC	LC	LC	x														x	x							
Myrmotherula behni	LC	LC	LC	x	x										x			x								
Myrmotherula menetriessi	LC	LC	LC	x														x	x							
Myrmotherula assimilis	LC	LC	LC	x														x								
Dichrozona cincta	LC	LC	LC	x														x	x					x		
Herpsilochmus dugandi	LC	LC	LC	x														x								
Herpsilochmus dorsimaculatus	LC	LC	LC	x														x	x							
Herpsilochmus axillaris	LC	LC	VU	x	x										x											
Herpsilochmus rufimarginatus	LC	LC	LC	x	x					x			x		x	x										
Microrhopias quixensis	LC	LC	LC	x	x								x		x	x		x	x							
Formicivora grisea	LC	LC	LC		x		x			x					x	x		x	x	x						
Drymophila devillei	LC	LC	LC	x	x										x			x								
Drymophila hellmayri	LC	LC	NT	x	x		x													x						
Drymophila klaguesi	LC	LC	LC		x										x											
Drymophila caudata	LC	LC	NT		x	x	x								x											
Drymophila striaticeps	LC	LC	LC		x	x									x											
Hypocnemis flavescens	LC	LC	LC	x														x	x							
Hypocnemis peruviana	LC	LC	LC	x														x								
Hypocnemis hypoxantha	LC	LC	LC	x														x								
Cercomacroides parkeri	LC	LC	LC		x										x											
Cercomacroides tyrannina	LC	LC	LC	x	x								x		x	x		x	x							
Cercomacroides serva	LC	LC	LC	x														x								
Cercomacroides nigrescens	LC	LC	LC	x	x										x			x								
Cercomacroides fuscicauda	LC	LC	LC	x														x								
Cercomacra cinerascens	LC	LC	LC	x														x	x							
Cercomacra nigricans	LC	LC	LC	x	x		x						x		x	x										
Pyriglena leuconota	LC	LC	LC	x	x										x											
Myrmoborus leucophrys	LC	LC	LC	x														x	x							
Myrmoborus lugubris	LC	LC	VU	x														x								
Myrmoborus myotherinus	LC	LC	LC	x														x								
Hypocnemoides melanopogon	LC	LC	LC	x														x	x							
Myrmochanes hemileucus	LC	LC	LC	x														x								
Gymnocichla nudiceps	LC	LC	LC	x			x						x			x										
Sclateria naevia	LC	LC	LC	x														x	x					x		
Percnostola rufifrons	LC	LC	LC	x														x								
Myrmelastes schistaceus	LC	LC	LC	x														x								
Myrmelastes leucostigma	LC	LC	LC	x	x										x			x	x							
Myrmeciza longipes	LC	LC	LC	x	x		x								x	x			x	x				x		
Poliocrania exsul	LC	LC	LC	x									x			x								x		
Sipia palliata	LC	LC	NT	x	x										x	x								x		
Sipia nigricauda	LC	LC	LC	x									x											x		
Sipia berlepschi	LC	LC	LC	x									x											x		
Akletos melanoceps	LC	LC	LC	x														x								
Hafferia fortis	LC	LC	NT	x														x						x		
Hafferia zeledoni	LC	LC	LC	x	x								x		x									x		
Hafferia inmaculata	LC	LC	LC	x	x										x									x		
Aprositornis disjuncta	LC	LC	LC	x															x							
Myrmophylax atrothorax	LC	LC	LC	x						x								x	x							
Ammonastes pelzelni	LC	LC	LC	x														x						x		
Myrmornis torquata	LC	LC	LC	x									x			x		x						x		
Pithys albifrons	LC	LC	LC	x	x										x			x	x							
Gymnopithys bicolor	LC	LC	LC	x									x			x					x					
Gymnopithys leucaspis	LC	LC	LC	x														x								
Rhegmatorhina cristata	LC	LC	LC	x														x								
Rhegmatorhina melanosticta	LC	LC	LC	x														x								
Hylophylax naevioides	LC	LC	LC	x	x								x		x	x										

Family and species	National category 2002	National category 2016	Global category 2018	ECOSYSTEMS									REGIONS										GROUPS CONSERVATION CONCERN			
				L. rain-forest	Sub-A. forest	High A. forest	L. dry forest	Par-amo	Man-grove	Sav-anna	Fwt.	C.wt	Pacific	P. Ocean	Andes	Caribbean	C. Sea	Amazon	Orinoquia	SNSM	Darién highlands	Sa&Pr.	Game-birds	T. forest insectiv.	Large Frugiv.	Forest Raptors
Hylophylax naevius	LC	LC	LC	x	x													x	x							
Hylophylax punctulatus	LC	LC	LC	x														x								
Willisornis poecilonotus	LC	LC	LC	x	x										x			x								
Phlegopsis nigromaculata	LC	LC	LC	x														x	x							
Phlegopsis erythroptera	LC	LC	LC	x														x								
Phaenostictus mcleannani	LC	LC	LC	x									x			x										
**Conopophagidae**																										
Pittasoma michleri	LC	LC	LC	x																	x			x		
Pittasoma rufopileatum	LC	LC	NT	x	x								x		x									x		
Conopophaga aurita	LC	LC	LC	x														x						x		
Conopophaga castaneiceps	LC	LC	LC	x	x								x		x									x		
**Grallaridae**																										
Grallaria squamigera	LC	LC	LC		x	x									x									x		
Grallaria gigantea	VU	VU	VU			x									x									x		
Grallaria alleni	EN	EN	VU		x										x									x		
Grallaria guatimalensis	LC	LC	LC	x	x								x		x					x	x			x		
Grallaria haplonota	LC	LC	LC	x	x								x		x									x		
Grallaria dignissima	LC	LC	LC	x														x						x		
Grallaria ruficapilla	LC	LC	LC		x	x									x									x		
Grallaria bangsi	VU	VU	VU		x															x				x		
Grallaria kaestneri	EN	EN	EN		x										x									x		
Grallaria rufocinerea	VU	VU	VU			x									x									x		
Grallaria nuchalis	LC	LC	LC		x	x									x									x		
Grallaria flavotincta	LC	LC	LC		x										x									x		
Grallaria hypoleuca	LC	LC	LC		x										x									x		
Grallaria rufula	LC	LC	LC		x	x									x					x				x		
Grallaria quitensis	LC	LC	LC			x		x							x									x		
Grallaria urraoensis	EN	EN	CR			x									x									x		
Grallaria milleri	EN	EN	VU			x									x									x		
Hylopezus perspicillatus	LC	LC	LC	x									x			x								x		
Hylopezus macularius	LC	LC	LC	x														x						x		
Hylopezus dives	LC	LC	LC	x	x								x		x									x		
Hylopezus fulviventris	LC	LC	LC	x														x						x		
Myrmothera campanisoma	LC	LC	LC	x														x						x		
Grallaricula flavirostris	LC	LC	NT	x	x										x						x			x		
Grallaricula cucullata	LC	LC	VU		x										x									x		
Grallaricula ferrugineipectus	LC	LC	LC	x	x										x					x				x		
Grallaricula nana	LC	LC	LC		x	x									x									x		
Grallaricula lineifrons	LC	LC	NT		x	x									x									x		
**Rhinocryptidae**																										
Liosceles thoracicus	LC	LC	LC	x														x						x		
Acropternyx orthonyx	LC	LC	LC		x	x									x									x		
Myornis senilis	LC	LC	LC		x	x									x									x		
Scytalopus latrans	LC	LC	LC		x	x									x									x		
Scytalopus sanctaemartae	VU	VU	LC		x															x				x		
Scytalopus micropterus	LC	LC	LC		x										x									x		
Scytalopus atratus	LC	LC	LC		x										x									x		
Scytalopus panamensis	VU	VU	VU		x																x			x		
Scytalopus chocoensis	LC	LC	LC	x	x								x		x									x		
Scytalopus rodriguezi	VU	VU	EN		x										x									x		
Scytalopus stilesi	VU	EN	LC		x										x									x		
Scytalopus alvarezlopezi	LC	LC	N/A*		x										x									x		
Scytalopus vicinior	LC	LC	LC		x										x									x		
Scytalopus latebricola	LC	LC	NT		x	x														x				x		
Scytalopus perijanus	VU	VU	VU		x	x		x							x									x		
Scytalopus spillmanni	LC	LC	LC		x	x									x									x		
Scytalopus griseicollis	LC	LC	LC		x	x									x									x		

Family and species	National category 2002	National category 2016	Global category 2018	ECOSYSTEMS L. rain-forest	Sub-A. forest	High A. forest	L. dry forest	Par-amo	Man-grove	Sav-anna	Fwt.	C.wt	REGIONS Pacific	P. Ocean	Andes	Caribbean	C. Sea	Amazon	Orinoquia	SNSM	Darién highlands	Sa&Pr.	GROUPS CONSERVATION CONCERN Game-birds	T. forest insectiv.	Large Frugiv.	Forest Raptors
Scytalopus canus	EN	EN	EN			x									x									x		
Scytalopus opacus	LC	LC	LC			x									x									x		
**Formicariidae**																										
Formicarius colma	LC	LC	LC	x														x	x					x		
Formicarius analis	LC	LC	LC	x	x		x						x		x	x		x						x		
Formicarius nigricapillus	LC	LC	LC	x	x								x		x									x		
Formicarius rufipectus	LC	LC	LC		x										x									x		
Chamaeza campanisoma	LC	LC	LC	x	x										x									x		
Chamaeza nobilis	LC	LC	LC	x														x						x		
Chamaeza turdina	LC	LC	LC		x	x									x									x		
Chamaeza mollissima	LC	LC	LC		x	x									x									x		
**Furnariidae**																										
Sclerurus mexicanus	LC	LC	LC	x	x								x		x			x						x		
Sclerurus rufigularis	LC	LC	LC	x	x										x			x						x		
Sclerurus guatemalensis	LC	LC	LC	x									x			x								x		
Sclerurus caudacutus	LC	LC	LC	x														x						x		
Sclerurus albigularis	LC	LC	NT	x	x										x					x				x		
Certhiasomus stictolaemus	LC	LC	LC	x														x								
Sittasomus griseicapillus	LC	LC	LC	x	x								x		x	x		x	x							
Deconychura longicauda	LC	LC	LC	x	x								x		x	x		x								
Dendrocincla tyrannina	LC	LC	LC		x	x									x											
Dendrocincla merula	LC	LC	LC	x														x	x							
Dendrocincla homochroa	LC	LC	LC	x	x								x		x					x						
Dendrocincla fuliginosa	LC	LC	LC	x	x								x		x	x		x	x	x	x					
Glyphorhynchus spirurus	LC	LC	LC	x	x								x		x	x		x	x		x					
Dendrexetastes rufigula	LC	LC	LC	x														x								
Nasica longirostris	LC	LC	LC	x														x								
Dendrocolaptes sanctithomae	LC	LC	LC	x									x			x										
Dendrocolaptes certhia	LC	LC	LC	x														x								
Dendrocolaptes picumnus	LC	LC	LC	x	x	x									x			x		x						
Hylexetastes stresemanni	LC	LC	LC	x														x								
Xiphocolaptes promeropirhynchus	LC	LC	LC	x	x	x									x	x		x	x	x						
Xiphorhynchus obsoletus	LC	LC	LC	x														x	x							
Xiphorhynchus ocellatus	LC	LC	LC	x														x	x							
Xiphorhynchus elegans	LC	LC	LC	x														x								
Xiphorhynchus susurrans	LC	LC	LC	x	x								x		x	x			x	x						
Xiphorhynchus guttatus	LC	LC	LC	x	x										x			x	x							
Xiphorhynchus lachrymosus	LC	LC	LC	x	x								x		x	x										
Xiphorhynchus erythropygius	LC	LC	LC	x	x								x		x	x					x					
Xiphorhynchus triangularis	LC	LC	LC		x	x									x											
Dendroplex picus	LC	LC	LC	x			x		x				x			x	x	x	x	x						
Dendroplex kienerii	LC	LC	NT	x														x								
Campylorhamphus	LC	LC	LC	x	x		x						x		x	x				x						
Campylorhamphus	LC	LC	LC	x														x								
Campylorhamphus pusillus	LC	LC	LC	x	x										x											
Drymotoxeres pucheranii	LC	LC	NT		x	x									x											
Lepidocolaptes souleyetii	LC	LC	LC	x	x		x						x		x	x			x	x						
Lepidocolaptes lacrymiger	LC	LC	LC		x	x									x					x						
Xenops tenuirostris	LC	LC	LC	x														x								
Xenops minutus	LC	LC	LC	x	x								x		x	x		x	x	x						
Xenops rutilans	LC	LC	LC		x	x									x					x	x					
Berlepschia rikeri	LC	LC	LC								x							x								
Microxenops milleri	LC	LC	LC	x														x	x							
Pseudocolaptes lawrencii	LC	LC	LC		x										x											
Pseudocolaptes boissonneautii	LC	LC	LC		x	x									x											
Premnornis guttuliger	LC	LC	LC		x	x									x											
Furnarius leucopus	LC	LC	LC	x			x						x			x		x								

Family and species	National category 2002	National category 2016	Global category 2018	ECOSYSTEMS									REGIONS										GROUPS CONSERVATION CONCERN			
				L. rain-forest	Sub-A. forest	High A. forest	L. dry forest	Par-amo	Man-grove	Sav-anna	Fwt.	C.wt	Pacific	P. Ocean	Andes	Caribbean	C. Sea	Amazon	Orinoquia	SNSM	Darién highlands	Sa&Pr.	Game-birds	T. forest insectiv.	Large Frugiv.	Forest Raptors
Furnarius torridus	LC	LC	LC	x														x								
Furnarius minor	LC	LC	LC	x														x								
Lochmias nematura	LC	LC	LC		x										x						x			x		
Cinclodes albidiventris	LC	LC	LC					x							x					x						
Cinclodes excelsior	LC	LC	LC					x							x											
Anabazenops dorsalis	LC	LC	LC	x	x										x			x								
Philydor fuscipenne	LC	LC	LC	x	x								x		x	x										
Philydor erythrocercum	LC	LC	LC	x	x										x			x								
Philydor erythropterum	LC	LC	LC	x														x								
Philydor rufum	LC	LC	LC		x										x											
Philydor pyrrhodes	LC	LC	LC	x														x								
Anabacerthia striaticollis	LC	LC	LC	x	x	x									x					x						
Anabacerthia variegaticeps	LC	LC	LC	x	x										x											
Anabacerthia ruficaudata	LC	LC	LC	x														x								
Syndactyla subalaris	LC	LC	LC	x	x	x									x						x					
Ancistrops strigilatus	LC	LC	LC	x														x								
Clibanornis rubiginosus	LC	LC	LC	x	x								x		x						x					
Clibanornis rufipectus	VU	VU	NT	x	x															x						
Thripadectes ignobilis	LC	LC	LC	x	x										x											
Thripadectes flammulatus	LC	LC	LC		x	x									x					x						
Thripadectes holostictus	LC	LC	LC		x	x									x											
Thripadectes virgaticeps	LC	LC	LC		x										x											
Thripadectes melanorhynchus	LC	LC	LC		x										x											
Automolus rufipileatus	LC	LC	LC	x														x								
Automolus melanopezus	LC	LC	LC	x														x								
Automolus ochrolaemus	LC	LC	LC	x	x								x		x	x		x								
Automolus subulatus	LC	LC	LC	x	x								x		x	x		x								
Automolus infuscatus	LC	LC	LC	x														x	x							
Premnoplex brunnescens	LC	LC	LC	x	x	x									x					x	x					
Margarornis stellatus	LC	LC	NT		x										x											
Margarornis bellulus	VU	VU	NT		x																x					
Margarornis squamiger	LC	LC	LC		x	x									x											
Leptasthenura andicola	LC	LC	LC					x							x					x						
Phacellodomus rufifrons	LC	LC	LC							x									x							
Hellmayrea gularis	LC	LC	LC			x									x											
Asthenes flammulata	LC	LC	LC					x							x											
Asthenes wyatti	LC	LC	LC					x							x					x						
Asthenes perijana	EN	EN	EN			x									x											
Asthenes fuliginosa	LC	LC	LC					x							x											
Metopothrix aurantiaca	LC	LC	LC	x														x								
Xenerpestes minlosi	LC	LC	LC	x			x						x			x										
Siptornis striaticollis	LC	LC	LC		x	x									x											
Thripophaga cherriei	LC	LC	VU	x															x							
Cranioleuca vulpina	LC	LC	LC	x															x							
Cranioleuca subcristata	LC	LC	LC	x	x										x	x			x							
Cranioleuca erythrops	LC	LC	LC		x										x						x					
Cranioleuca hellmayri	LC	LC	LC		x	x														x						
Cranioleuca curtata	LC	LC	VU	x	x										x											
Cranioleuca gutturata	LC	LC	LC	x														x								
Certhiaxis cinnamomeus	LC	LC	LC								x					x		x	x							
Certhiaxis mustelinus	LC	LC	LC								x							x								
Synallaxis gujanensis	LC	LC	LC	x	x										x			x								
Synallaxis brachyura	LC	LC	LC	x	x								x		x	x					x					
Synallaxis subpudica	LC	LC	LC		x	x									x											
Synallaxis moesta	LC	LC	NT	x	x										x			x	x							
Synallaxis albigularis	LC	LC	LC	x	x										x			x								
Synallaxis albescens	LC	LC	LC	x	x		x			x			x		x	x		x	x	x						

Family and species	National category 2002	National category 2016	Global category 2018	L. rain-forest	Sub-A. forest	High A. forest	L. dry forest	Par-amo	Man-grove	Sav-anna	Fwt.	C.wt	Pacific	P. Ocean	Andes	Caribbean	C. Sea	Amazon	Orinoquia	SNSM	Darién highlands	Sa&Pr.	Game-birds	T. forest insectiv.	Large Frugiv.	Forest Raptors
				ECOSYSTEMS									REGIONS										GROUPS CONSERVATION CONCERN			
Synallaxis azarae	LC	LC	LC		x	x									x											
Synallaxis candei	LC	LC	LC				x		x							x	x			x						
Synallaxis fuscorufa	NT	NT	VU		x	x														x						
Synallaxis unirufa	LC	LC	LC		x	x									x											
Synallaxis cinnamomea	LC	LC	LC	x	x		x								x											
Synallaxis rutilans	LC	LC	LC	x														x	x							
Synallaxis cherriei	DD	DD	NT	x														x								
**Tyrannidae**																										
Phyllomyias burmeisteri	LC	LC	LC	x	x										x					x						
Phyllomyias griseiceps	LC	LC	LC	x	x		x						x		x					x						
Phyllomyias nigrocapillus	LC	LC	LC		x	x									x					x						
Phyllomyias cinereiceps	LC	LC	LC		x	x									x											
Phyllomyias uropygialis	LC	LC	LC		x	x									x											
Phyllomyias plumbeiceps	LC	LC	LC		x										x											
Tyrannulus elatus	LC	LC	LC	x	x		x						x		x	x		x	x	x						
Myiopagis gaimardii	LC	LC	LC	x	x								x		x	x		x	x	x						
Myiopagis caniceps	LC	LC	LC	x			x						x					x								
Myiopagis viridicata	LC	LC	LC	x	x		x						x		x	x				x						
Elaenia flavogaster	LC	LC	LC	x	x		x						x		x	x		x	x	x						
Elaenia martinica	LC	LC	LC	x													x					x				
Elaenia spectabilis	LC	LC	LC	x														x								
Elaenia albiceps	LC	LC	LC	x		x									x			x								
Elaenia parvirostris	LC	LC	LC	x	x										x	x		x	x							
Elaenia strepera	LC	LC	LC	x														x	x							
Elaenia gigas	LC	LC	LC	x														x								
Elaenia cristata	LC	LC	LC							x								x	x							
Elaenia chiriquensis	LC	LC	LC		x					x					x	x		x	x	x						
Elaenia brachyptera	LC	LC	LC	x	x										x											
Elaenia ruficeps	LC	LC	LC							x								x	x							
Elaenia frantzii	LC	LC	LC		x	x									x					x						
Elaenia pallantagae	LC	LC	LC		x	x									x											
Ornithion brunneicapillus	LC	LC	LC	x	x								x		x	x				x						
Ornithion inerme	LC	LC	LC	x														x	x							
Camptostoma obsoletum	LC	LC	LC	x	x		x						x		x	x		x	x	x						
Mecocerculus poecilocercus	LC	LC	LC		x	x									x											
Meceocerculus stictopterus	LC	LC	LC		x	x									x											
Meceocerculus leucophrys	LC	LC	LC			x									x					x						
Mecocerculus minor	LC	LC	LC		x	x									x											
Anairetes parulus	LC	LC	LC		x	x									x											
Uromyias agilis	LC	LC	LC			x									x											
Serpophaga cinerea	LC	LC	LC								x				x					x						
Serpophaga hypoleuca	LC	LC	LC							x								x	x							
Phaeomyias murina	LC	LC	LC		x		x								x	x		x	x	x						
Capsiempis flaveola	LC	LC	LC	x			x						x		x	x		x	x	x						
Polystictus pectoralis	NT	VU	NT							x									x							
Pseudocolopteryx acutipennis	CR	CR	LC								x				x											
Pseudotriccus pelzelni	LC	LC	LC	x	x										x						x					
Pseudotriccus ruficeps	LC	LC	LC		x	x									x											
Corythopis torquatus	LC	LC	LC	x														x	x					x		
Euscarthmus meloryphus	LC	LC	LC				x						x			x										
Stigmatura napensis	LC	LC	LC	x														x	x							
Zimmerius villisimus	LC	LC	LC	x	x										x					x	x					
Zimmerius albigularis	LC	LC	LC	x									x													
Zimmerius gracilipes	LC	LC	LC	x														x	x							
Zimmerius chrysops	LC	LC	LC	x	x										x					x						
Phylloscartes poecilotis	LC	LC	LC		x										x											
Phylloscartes ophthalmicus	LC	LC	LC		x										x											

Family and species	National category 2002	National category 2016	Global category 2018	ECOSYSTEMS L. rain-forest	Sub-A. forest	High A. forest	L. dry forest	Par-amo	Man-grove	Sav-anna	Fwt.	C.wt	REGIONS Pacific	P. Ocean	Andes	Caribbean	C. Sea	Amazon	Orinoquia	SNSM	Darién highlands	Sa&Pr.	GROUPS CONSERVATION CONCERN Game-birds	T. forest insectiv.	Large Frugiv.	Forest Raptors
Phylloscartes lanyoni	EN	EN	EN	x												x										
Phylloscartes orbitalis	LC	LC	LC	x	x										x			x								
Phylloscartes superciliaris	LC	LC	LC		x										x						x					
Mionectes striaticollis	LC	LC	LC		x	x									x											
Mionectes olivaceus	LC	LC	LC	x	x								x		x	x				x	x					
Mionectes oleagineus	LC	LC	LC	x	x								x		x	x		x	x	x						
Leptopogon amaurocephalus	LC	LC	LC	x	x										x	x		x	x	x						
Leptopogon superciliaris	LC	LC	LC	x	x										x											
Leptopogon rufipectus	LC	LC	LC		x	x									x											
Sublegatus arenarum	LC	LC	LC				x		x							x	x		x							
Sublegatus obscurior	LC	LC	LC	x														x								
Inezia tenuirostris	LC	LC	LC				x									x										
Inezia subflava	LC	LC	LC	x						x								x	x							
Inezia caudata	LC	LC	LC				x			x						x			x							
Myiotriccus ornatus	LC	LC	LC	x	x										x											
Myiornis atricapillus	LC	LC	LC	x									x			x										
Myiornis ecaudatus	LC	LC	LC	x														x	x							
Oncostoma cinereigulare	LC	LC	LC	x									x													
Oncostoma olivaceum	LC	LC	LC	x	x		x						x		x	x				x						
Lophotriccus pileatus	LC	LC	LC	x	x										x						x					
Lophotriccus vitiosus	LC	LC	LC	x														x								
Lophotriccus galeatus	LC	LC	LC	x														x	x							
Atalotriccus pilaris	LC	LC	LC		x		x								x	x			x	x						
Hemitriccus zosterops	LC	LC	LC	x														x	x							
Hemitriccus iohannis	LC	LC	LC	x														x								
Hemitriccus striaticollis	LC	LC	LC	x														x								
Hemitriccus margaritaceiventer	LC	LC	LC				x									x		x		x						
Hemitriccus granadensis	LC	LC	LC		x	x									x					x						
Poecilotriccus ruficeps	LC	LC	LC		x	x									x											
Poecilotriccus capitalis	LC	LC	LC	x														x								
Poecilotriccus latirostris	LC	LC	LC	x														x								
Poecilotriccus sylvia	LC	LC	LC	x	x		x								x	x			x							
Poecilotriccus calopterus	LC	LC	LC	x	x										x			x								
Todirostrum maculatum	LC	LC	LC	x														x								
Todirostrum cinereum	LC	LC	LC	x	x		x			x			x		x	x			x	x						
Todirostrum nigriceps	LC	LC	LC	x									x			x										
Todirostrum chrysocrotaphum	LC	LC	LC	x														x								
Cnipodectes subbrunneus	LC	LC	LC	x									x			x		x								
Rhynchocyclus olivaceus	LC	LC	LC	x	x								x		x	x		x		x						
Rhynchocyclus brevirostris	LC	LC	LC	x	x								x								x					
Rhynchocyclus pacificus	LC	LC	LC	x	x								x		x											
Rhynchocyclus fulvipectus	LC	LC	LC	x	x										x											
Tolmomyias sulphurescens	LC	LC	LC	x	x		x			x			x		x	x		x	x	x						
Tolmomyias traylori	LC	LC	LC	x														x								
Tolmomyias assimilis	LC	LC	LC	x	x								x		x	x		x	x							
Tolmomyias poliocephalus	LC	LC	LC	x														x	x							
Tolmomyias flaviventris	LC	LC	LC	x			x						x			x		x	x	x						
Neopipo cinnamomea	LC	LC	LC	x														x	x							
Platyrinchus saturatus	LC	LC	LC	x														x								
Platyrinchus mystaceus	LC	LC	LC	x	x										x					x	x					
Platyrinchus coronatus	LC	LC	LC	x									x			x		x								
Platyrinchus flavigularis	LC	LC	LC		x										x											
Platyrinchus platyrhynchos	LC	LC	LC	x														x	x							
Onycorhynchus coronatus	LC	LC	LC	x									x			x		x	x	x						
Myiophobus flavicans	LC	LC	LC		x	x									x											
Myiophobus phoenicomitra	LC	LC	LC	x	x										x											
Myiophobus fasciatus	LC	LC	LC	x	x		x						x		x				x	x						

Family and species	National category 2002	National category 2016	Global category 2018	ECOSYSTEMS									REGIONS										GROUPS CONSERVATION CONCERN			
				L. rain-forest	Sub-A. forest	High A. forest	L. dry forest	Par-amo	Man-grove	Sav-anna	Fwt.	C.wt	Pacific	P. Ocean	Andes	Caribbean	C. Sea	Amazon	Orinoquia	SNSM	Darién highlands	Sa&Pr.	Game-birds	T. forest insectiv.	Large Frugiv.	Forest Raptors
Myiobius villosus	LC	LC	LC	x	x										x						x					
Myiobius barbatus	LC	LC	LC	x									x			x		x								
Myiobius atricaudus	LC	LC	LC	x	x		x						x		x	x		x								
Terenotriccus erythrurus	LC	LC	LC	x	x								x		x	x		x	x							
Pyrrhomyias cinnamomea	LC	LC	LC		x	x									x					x						
Hirundinea ferruginea	LC	LC	LC	x	x										x			x	x	x						
Nephelomyias pulcher	LC	LC	LC		x										x											
Lathotriccus euleri	LC	LC	LC	x												x		x	x							
Aphanotriccus audax	NT	NT	NT	x												x										
Cnemotriccus fuscatus	LC	LC	LC	x			x									x		x	x	x						
Empidonax virescens	LC	LC	LC	x	x	x							x		x	x				x	x	x				
Empidonax traillii	LC	LC	LC	x	x		x						x		x	x		x	x	x		x				
Empidonax alnorum	LC	LC	LC	x	x		x						x		x			x	x	x	x	x				
Contopus cooperi	NT	NT	NT		x	x									x					x		x				
Contopus fumigatus	LC	LC	LC	x	x										x											
Contopus sordidulus	LC	LC	LC	x	x	x	x						x		x	x				x	x	x				
Contopus virens	LC	LC	LC	x	x		x						x		x	x		x	x	x	x	x				
Contopus cinereus	LC	LC	LC		x										x					x						
Mitrephanes phaeocercus	LC	LC	LC	x									x								x					
Sayornis nigricans	LC	LC	LC								x				x					x						
Pyrocephalus rubinus	LC	LC	LC	x	x	x	x			x			x		x	x		x	x	x						
Knipolegus orenocensis	LC	LC	LC	x														x	x							
Knipolegus poecilurus	LC	LC	LC		x										x			x								
Knipolegus poecilocercus	LC	LC	LC	x														x	x							
Ochthornis littoralis	LC	LC	LC								x							x	x							
Muscisaxicola maculirostris	EN	EN	LC							x					x											
Muscisaxicola alpinus	LC	LC	LC					x							x											
Agriornis montanus	LC	LC	LC					x							x											
Myiotheretes striaticollis	LC	LC	LC		x	x									x					x						
Myiotheretes pernix	EN	EN	EN		x	x														x						
Myiotheretes fumigatus	LC	LC	LC		x	x									x					x						
Cnemarchus erythropygius	LC	LC	LC			x		x							x					x						
Fluvicola pica	LC	LC	LC								x				x	x		x	x							
Fluvicola nengeta	LC	LC	LC								x		x													
Arundinicola leucocephala	LC	LC	LC								x					x		x	x							
Ochthoeca frontalis	LC	LC	LC		x	x									x											
Ochthoeca diadema	LC	LC	LC		x	x									x					x						
Ochthoeca cinnamomeiventris	LC	LC	LC		x	x									x											
Ochthoeca rufipectoralis	LC	LC	LC		x	x									x					x						
Ochthoeca fumicolor	LC	LC	LC					x							x											
Colonia colonus	LC	LC	LC	x	x								x		x	x		x								
Machetornis rixosa	LC	LC	LC				x			x			x		x	x		x	x	x						
Legatus leucophaius	LC	LC	LC	x	x								x		x	x		x	x	x						
Myiozetetes cayanensis	LC	LC	LC	x	x					x			x		x	x		x	x	x						
Myiozetetes similis	LC	LC	LC	x	x		x								x	x		x	x	x						
Myiozetetes granadensis	LC	LC	LC	x	x								x		x	x		x	x							
Myiozetetes luteiventris	LC	LC	LC	x														x								
Phelpsia inornata	LC	LC	LC							x									x							
Pitangus sulphuratus	LC	LC	LC	x	x		x			x			x		x	x		x	x	x						
Pitangus lictor	LC	LC	LC						x		x		x		x	x	x	x	x							
Conopias albovittatus	LC	LC	LC	x									x													
Conopias parvus	LC	LC	LC	x														x	x							
Conopias cinchoneti	LC	LC	VU	x	x										x											
Myiodinastes chrysocephalus	LC	LC	LC		x	x									x					x	x					
Myiodinastes maculatus	LC	LC	LC	x	x								x		x	x		x	x	x						
Megarhynchus pitangua	LC	LC	LC	x	x								x		x	x		x	x	x	x					
Tyrannopsis sulphurea	LC	LC	LC	x						x								x	x							

				ECOSYSTEMS									REGIONS										GROUPS CONSERVATION CONCERN			
**Family and species**	**National category 2002**	**National category 2016**	**Global category 2018**	**L. rain-forest**	**Sub-A. forest**	**High A. forest**	**L. dry forest**	**Par-amo**	**Man-grove**	**Sav-anna**	**Fwt.**	**C.wt**	**Pacific**	**P. Ocean**	**Andes**	**Caribbean**	**C. Sea**	**Amazon**	**Orinoquia**	**SNSM**	**Darién highlands**	**Sa&Pr.**	**Game-birds**	**T. forest insectiv.**	**Large Frugiv.**	**Forest Raptors**
Empidonomus varius	LC	LC	LC	x						x						x		x	x							
Empidonomus	LC	LC	LC	x									x		x	x		x	x	x						
Tyrannus melancholicus	LC	LC	LC	x	x	x	x			x			x		x	x		x	x	x						
Tyrannus savana	LC	LC	LC	x	x	x	x			x			x		x			x	x	x						
Tyrannus tyrannus	LC	LC	LC	x	x	x	x						x		x	x		x	x	x						
Tyrannus dominicensis	LC	LC	LC				x			x			x		x	x			x	x		x				
Rhytipterna holerythra	LC	LC	LC	x	x								x		x	x					x					
Rhytipterna simplex	LC	LC	LC	x														x	x							
Rhytipterna immunda	LC	LC	LC	x						x								x								
Sirystes albogriseus	LC	LC	LC	x									x													
Sirystes albocinereus	LC	LC	LC	x														x	x							
Myiarchus tuberculifer	LC	LC	LC	x	x		x						x		x	x		x	x	x	x					
Myiarchus swainsoni	LC	LC	LC	x														x	x							
Myiarchus venezuelensis	LC	LC	LC				x									x			x	x						
Myiarchus panamensis	LC	LC	LC				x		x				x	x		x	x			x						
Myiarchus ferox	LC	LC	LC	x														x	x							
Myiarchus apicalis	LC	LC	LC		x		x								x	x										
Myiarchus cephalotes	LC	LC	LC		x	x									x											
Myiarchus crinitus	LC	LC	LC	x	x	x	x						x		x	x				x		x				
Myiarchus tyrannulus	LC	LC	LC				x		x							x	x		x	x						
Ramphotrigon megacephalum	LC	LC	LC	x														x	x							
Ramphotrigon ruficauda	LC	LC	LC	x														x	x							
Ramphotrigon fuscicauda	LC	LC	LC	x														x								
Attila cinnamomeus	LC	LC	LC	x														x	x							
Attila torridus	VU	VU	VU	x									x													
Attila citriniventris	LC	LC	LC	x														x								
Attila bolivianus	LC	LC	LC	x														x								
Attila spadiceus	LC	LC	LC	x	x								x		x	x		x	x	x	x					
**Oxyruncidae**																										
Oxyruncus cristatus	LC	LC	LC	x	x											x					x					
**Cotingidae**																										
Pipreola riefferii	LC	LC	LC		x	x									x											
Pipreola arcuata	LC	LC	LC		x	x									x											
Pipreola aureopectus	LC	LC	LC	x	x										x					x						
Pipreola jucunda	LC	LC	LC	x	x										x											
Pipreola lubomirskii	LC	LC	LC		x										x											
Pipreola chlorolepidota	VU	NT	NT		x										x											
Ampelioides tschudii	LC	LC	LC	x	x	x									x											
Doliornis remseni	EN	EN	VU			x									x											
Ampelion rubrocristatus	LC	LC	LC		x	x									x					x						
Ampelion rufaxilla	VU	VU	LC		x	x									x											
Phoenicircus nigricollis	LC	LC	LC	x														x								
Rupicola rupicola	LC	LC	LC	x														x	x						x	
Rupicola peruvianus	LC	LC	LC	x	x										x										x	
Snowornis subalaris	LC	LC	NT	x	x										x			x								
Snowornis cryptolophus	LC	LC	LC	x	x										x											
Querula purpurata	LC	LC	LC	x	x								x		x	x		x	x						x	
Pyroderus scutatus	VU	VU	LC		x	x									x										x	
Cephalopterus ornatus	LC	LC	LC	x	x										x			x	x						x	
Cephalopterus penduliger	EN	EN	VU	x	x								x		x										x	
Perissocephalus tricolor	LC	LC	LC	x														x	x						x	
Cotinga nattererii	LC	LC	LC	x	x								x		x	x										
Cotinga maynana	LC	LC	LC	x														x								
Cotinga cotinga	LC	LC	LC	x														x	x							
Cotinga cayana	LC	LC	LC	x														x	x							
Lipaugus weberi	CR	CR	CR		x										x											
Lipaugus fuscocinereus	LC	LC	LC		x	x									x										x	

				ECOSYSTEMS									REGIONS										GROUPS CONSERVATION CONCERN			
**Family and species**	**National category 2002**	**National category 2016**	**Global category 2018**	**L. rain-forest**	**Sub-A. forest**	**High A. forest**	**L. dry forest**	**Par-amo**	**Man-grove**	**Sav-anna**	**Fwt.**	**C.wt**	**Pacific**	**P. Ocean**	**Andes**	**Caribbean**	**C. Sea**	**Amazon**	**Orinoquia**	**SNSM**	**Darién highlands**	**Sa&Pr.**	**Game-birds**	**T. forest insectiv.**	**Large Frugiv.**	**Forest Raptors**
Lipaugus unirufus	LC	LC	LC	x	x								x		x	x										
Lipaugus vociferans	LC	LC	LC	x														x	x							
Procnias averano	LC	LC	LC	x												x									x	
Porphyrolaema porphyrolaema	LC	LC	LC	x														x								
Carpodectes hopkei	LC	LC	LC	x					x				x	x											x	
Xipholena punicea	LC	LC	LC	x														x	x							
Gymnoderus foetidus	LC	LC	LC	x														x	x						x	
**Pipridae**																										
Tyranneutes stolzmanni	LC	LC	LC	x														x	x							
Neopelma chrysocephalum	LC	LC	LC	x														x								
Chloropipo flavicapilla	VU	VU	VU		x										x											
Chiroxiphia lanceolata	LC	LC	LC	x			x									x				x						
Chiroxiphia pareola	LC	LC	LC	x														x								
Masius chrysopterus	LC	LC	LC	x	x										x											
Corapipo altera	LC	LC	N/A*	x									x								x					
Corapipo leucorrhoa	LC	LC	LC	x	x										x	x										
Xenopipo atronitens	LC	LC	LC	x														x	x							
Cryptopipo holochlora	LC	LC	LC	x	x								x		x			x	x							
Lepidothrix coronata	LC	LC	LC	x	x								x		x	x		x	x							
Lepidothrix isidorei	LC	LC	NT	x	x										x			x	x							
Heterocercus flavivertex	LC	LC	LC	x														x	x							
Manacus manacus	LC	LC	LC	x	x								x		x	x		x	x	x						
Pipra filicauda	LC	LC	LC	x												x		x	x							
Machaeropterus deliciosus	LC	LC	LC	x	x										x											
Machaeropterus regulus	LC	LC	LC	x	x										x	x		x	x							
Dixiphia pipra	LC	LC	LC	x	x										x	x		x	x							
Ceratopipra mentalis	LC	LC	LC	x									x													
Ceratopipra erythrocephala	LC	LC	LC	x	x								x		x	x		x	x	x						
**Tityridae**																										
Tityra inquisitor	LC	LC	LC	x	x								x		x	x		x	x	x						
Tityra cayana	LC	LC	LC	x	x										x	x		x	x							
Tityra semifasciata	LC	LC	LC	x	x								x		x	x				x						
Schiffornis major	LC	LC	LC	x														x								
Schiffornis veraepacis	LC	LC	LC	x	x								x		x											
Schiffornis stenorhyncha	LC	LC	LC	x	x								x		x	x				x						
Schiffornis turdina	LC	LC	LC	x	x										x			x	x							
Laniocera rufescens	LC	LC	LC	x									x			x					x					
Laniocera hypopyrra	LC	LC	LC	x						x								x	x							
Iodopleura isabellae	LC	LC	LC	x														x	x							
Laniisoma elegans	LC	LC	NT	x	x										x			x	x							
Pachyramphus versicolor	LC	LC	LC		x	x									x											
Pachyramphus rufus	LC	LC	LC	x	x		x								x	x			x	x						
Pachyramphus cinnamomeus	LC	LC	LC	x	x								x		x	x			x	x						
Pachyramphus castaneus	LC	LC	LC	x														x								
Pachyramphus polychopterus	LC	LC	LC	x	x								x		x	x		x	x	x						
Pachyramphus albogriseus	LC	LC	LC	x	x		x								x					x						
Pachyramphus marginatus	LC	LC	LC	x														x	x							
Pachyramphus homochrous	LC	LC	LC	x			x						x			x				x						
Pachyramphus minor	LC	LC	LC	x														x	x							
**Incertae Sedis**																										
Piprites chloris	LC	LC	LC	x	x										x	x		x	x							
**OSCINES**																										
**Vireonidae**																										
Cyclarhis gujanensis	LC	LC	LC	x	x		x								x	x		x	x	x						
Cyclarhis nigrirostris	LC	LC	LC		x	x									x											
Hylophilus flavipes	LC	LC	LC		x		x								x	x			x	x						
Hylophilus brunneiceps	LC	LC	LC	x														x	x							

Family and species	National category 2002	National category 2016	Global category 2018	ECOSYSTEMS L. rain-forest	Sub-A. forest	High A. forest	L. dry forest	Par-amo	Man-grove	Sav-anna	Fwt.	C.wt	REGIONS Pacific	P. Ocean	Andes	Caribbean	C. Sea	Amazon	Orinoquia	SNSM	Darién highlands	Sa&Pr.	GROUPS CONSERVATION CONCERN Game-birds	T. forest insectiv.	Large Frugiv.	Forest Raptors
Hylophilus thoracicus	LC	LC	LC	x														x								
Vireolanius eximius	LC	LC	LC	x	x										x	x					x					
Vireolanius leucotis	LC	LC	LC	x	x										x			x								
Tunchiornis ochraceiceps	LC	LC	LC	x									x					x	x							
Pachysylvia decurtata	LC	LC	LC	x									x			x										
Pachysylvia hypoxantha	LC	LC	LC	x														x								
Pachysylvia aurantiifrons	LC	LC	LC	x			x						x			x			x	x						
Pachysylvia semibrunnea	LC	LC	LC	x	x	x									x	x										
Vireo flavifrons	LC	LC	LC	x	x	x							x		x	x				x	x	x				
Vireo masteri	VU	VU	EN		x										x											
Vireo approximans	VU	VU	LC				x		x								x					x				
Vireo caribaeus	VU	VU	VU				x		x								x					x				
Vireo leucophrys	LC	LC	LC		x	x									x					x						
Vireo olivaceus	LC	LC	LC	x	x	x									x	x		x	x	x	x	x				
Vireo flavoviridis	LC	LC	LC	x	x	x							x		x	x		x	x	x	x	x				
Vireo altiloquus	LC	LC	LC	x	x								x		x	x		x	x	x	x	x				
**Corvidae**																										
Cyanolyca armillata	LC	LC	LC		x	x									x										x	
Cyanolyca turcosa	LC	LC	LC		x	x									x										x	
Cyanolyca pulchra	VU	VU	NT		x										x											
Cyanocorax violaceus	LC	LC	LC	x	x										x			x	x						x	
Cyanocorax affinis	LC	LC	LC	x	x		x						x		x	x				x					x	
Cyanocorax heilprini	LC	LC	LC	x						x								x							x	
Cyanocorax yncas	LC	LC	LC	x	x	x									x											
**Alaudidae**																										
Eremophila alpestris	EN	EN	LC							x					x											
**Hirundinidae**																										
Pygochelidon cyanoleuca	LC	LC	LC	x	x	x	x			x			x		x	x		x	x	x						
Pygochelidon melanoleuca	LC	LC	LC								x							x	x							
Orochelidon murina	LC	LC	LC		x	x				x					x					x						
Orochelidon flavipes	LC	LC	LC		x	x									x											
Atticora fasciata	LC	LC	LC								x							x	x							
Atticora tibialis	LC	LC	LC	x	x								x		x	x		x								
Stelgidopteryx ruficollis	LC	LC	LC	x	x		x						x		x	x		x	x	x	x					
Progne tapera	LC	LC	LC	x	x	x	x			x			x		x	x		x	x	x						
Progne subis	LC	LC	LC					x		x			x		x	x		x	x	x	x					
Progne chalybea	LC	LC	LC	x	x								x		x	x		x	x	x	x					
Tachycineta albiventer	LC	LC	LC								x		x			x		x	x							
Riparia riparia	LC	LC	LC					x		x			x		x	x		x	x	x	x	x				
Hirundo rustica	LC	LC	LC					x		x			x		x	x		x	x	x	x	x				
Petrochelidon pyrrhonota	LC	LC	LC					x		x			x		x	x		x	x	x	x	x				
**Troglodytidae**																										
Microcerculus marginatus	LC	LC	LC	x	x								x		x	x		x	x	x				x		
Odontorchilus branickii	LC	LC	LC	x	x										x											
Troglodytes aedon	LC	LC	LC	x	x	x	x	x					x		x	x		x	x	x						
Troglodytes solstitialis	LC	LC	LC		x	x									x											
Trogolodytes monticola	EN	EN	CR			x														x						
Cistothorus platensis	LC	LC	LC					x			x				x					x						
Cistothorus apolinari	EN	CR	EN								x				x											
Campylorhynchus	LC	LC	LC	x	x								x		x											
Campylorhynchus zonatus	LC	LC	LC	x	x		x						x		x	x										
Campylorhynchus nuchalis	LC	LC	LC				x									x			x							
Campylorhynchus griseus	LC	LC	LC		x		x			x			x		x	x			x	x						
Campylorhynchus turdinus	LC	LC	LC	x	x										x			x								
Pheugopedius spadix	LC	LC	LC	x	x										x						x					
Pheugopedius fasciatoventris	LC	LC	LC	x	x								x		x	x										
Pheugopedius euophrys	LC	LC	LC		x	x									x											

Family and species	National category 2002	National category 2016	Global category 2018	ECOSYSTEMS L. rain-forest	Sub-A. forest	High A. forest	L. dry forest	Par-amo	Man-grove	Sav-anna	Fwt.	C.wt	REGIONS Pacific	P. Ocean	Andes	Caribbean	C. Sea	Amazon	Orinoquia	SNSM	Darién highlands	Sa&Pr.	GROUPS CONSERVATION CONCERN Game-birds	T. forest insectiv.	Large Frugiv.	Forest Raptors
Pheugopedius mystacalis	LC	LC	LC		x										x											
Pheugopedius coraya	LC	LC	LC	x														x								
Pheugopedius rutilus	LC	LC	LC	x	x		x								x					x						
Pheugopedius sclateri	LC	LC	LC		x		x								x											
Thryophilus rufalbus	LC	LC	LC				x									x			x	x						
Thryophilus sernai	EN	EN	EN				x									x										
Thryophilus nicefori	CR	CR	CR		x										x											
Cantorchilus leucopogon	LC	LC	LC	x					x				x	x		x										
Cantorchilus nigricapillus	LC	LC	LC	x	x				x				x	x	x	x					x					
Cantorchilus leucotis	LC	LC	LC	x			x		x				x			x	x	x	x	x						
Cinnycerthia unirufa	LC	LC	LC		x	x									x											
Cinnycerthia olivascens	LC	LC	LC		x	x									x											
Henicorhina leucosticta	LC	LC	LC	x	x								x		x	x		x	x		x					
Henicorhina leucophrys	LC	LC	LC		x	x									x					x	x					
Henicorhina anachoreta	LC	LC	NT			x														x						
Henicorhina negreti	VU	VU	VU		x										x											
Cyphorhinus thoracicus	LC	LC	LC		x	x									x									x		
Cyphorhinus phaeocephalus	LC	LC	LC	x	x								x		x	x								x		
Cyphorhinus arada	LC	LC	LC	x	x										x			x						x		
**Polioptilidae**																										
Microbates collaris	LC	LC	LC	x	x										x			x						x		
Microbates cinereiventris	LC	LC	LC	x	x								x		x	x		x						x		
Ramphocaenus melanurus	LC	LC	LC	x	x								x		x	x		x	x	x						
Polioptila plumbea	LC	LC	LC	x	x		x						x		x	x		x	x	x						
Polioptila schistaceigula	LC	LC	LC	x									x			x					x					
**Donacobiidae**																										
Donacobius atricapilla	LC	LC	LC								x		x		x	x		x	x							
**Cinclidae**																										
Cinclus leucocephalus	LC	LC	LC								x				x					x						
**Turdidae**																										
Myadestes coloratus	VU	VU	LC		x																x					
Myadestes ralloides	LC	LC	LC	x	x	x									x											
Catharus aurantiirostris	LC	LC	LC	x	x										x					x						
Catharus fuscater	LC	LC	LC	x	x	x									x					x	x					
Catharus dryas	LC	LC	LC	x	x										x											
Catharus fuscescens	LC	LC	LC	x	x	x							x		x	x		x	x	x		x				
Catharus minimus	LC	LC	LC	x	x	x	x						x		x	x		x	x	x	x	x				
Catharus ustulatus	LC	LC	LC	x	x	x	x						x		x	x		x	x	x	x	x				
Entomodestes coracinus	LC	LC	LC	x	x	x									x											
Cichlopsis leucogenys	LC	LC	EN	x	x								x		x											
Turdus leucops	LC	LC	LC		x										x					x						
Turdus flavipes	LC	LC	LC	x	x										x					x						
Turdus leucomelas	LC	LC	LC	x	x		x								x	x			x	x						
Turdus fumigatus	LC	LC	LC	x	x										x	x		x	x							
Turdus hauxwelli	LC	LC	LC	x														x								
Turdus obsoletus	LC	LC	LC	x	x								x		x	x					x					
Turdus grayi	LC	LC	LC		x		x									x				x						
Turdus nudigenis	LC	LC	LC	x	x										x	x			x							
Turdus sanchezorum	LC	LC	LC	x														x								
Turdus lawrencii	LC	LC	LC	x														x	x							
Turdus ignobilis	LC	LC	LC	x	x	x									x	x		x	x							
Turdus fulviventris	LC	LC	LC		x										x											
Turdus olivater	LC	LC	LC	x	x										x					x						
Turdus fuscater	LC	LC	LC		x	x		x							x					x					x	
Turdus serranus	LC	LC	LC		x	x									x											
Turdus assimilis	LC	LC	LC	x									x								x					
Turdus albicollis	LC	LC	LC	x	x										x			x	x	x						

				ECOSYSTEMS									REGIONS										GROUPS CONSERVATION CONCERN			
**Family and species**	**National category 2002**	**National category 2016**	**Global category 2018**	**L. rain-forest**	**Sub-A. forest**	**High A. forest**	**L. dry forest**	**Par-amo**	**Man-grove**	**Sav-anna**	**Fwt.**	**C.wt**	**Pacific**	**P. Ocean**	**Andes**	**Caribbean**	**C. Sea**	**Amazon**	**Orinoquia**	**SNSM**	**Darién highlands**	**Sa&Pr.**	**Game-birds**	**T. forest insectiv.**	**Large Frugiv.**	**Forest Raptors**
**Mimidae**																										
Mimus gilvus	LC	LC	LC		x		x						x		x	x		x	x	x		x				
**Motacillidae**																										
Anthus lutescens	LC	LC	LC							x									x							
Anthus bogotensis	LC	LC	LC					x		x					x											
**Thraupidae**																										
Paroaria nigrogenis	LC	LC	LC								x				x	x			x							
Paroaria gularis	LC	LC	LC								x				x			x								
Schistochlamys melanopis	LC	LC	LC							x					x	x		x	x							
Cissopis leverianus	LC	LC	LC	x	x										x			x	x							
Sericossypha albocristata	LC	LC	VU		x	x									x										x	
Nemosia pileata	LC	LC	LC	x			x									x		x	x							
Creurgops verticalis	VU	VU	LC		x	x									x											
Kleinothraupis atropileus	LC	LC	LC		x	x									x											
Thlypopsis superciliaris	LC	LC	LC		x	x									x											
Sphenopsis frontalis	LC	LC	LC		x	x									x											
Sphenopsis melanotis	LC	LC	LC		x	x									x											
Pseudospingus verticalis	LC	LC	LC			x									x											
Cnemoscopus rubrirostris	LC	LC	LC		x	x									x											
Thlypopsis fulviceps	LC	LC	LC		x										x					x						
Thlypopsis ornata	NT	NT	LC		x	x									x											
Thlypopsis sordida	LC	LC	LC	x														x	x							
Eucometis penicillata	LC	LC	LC	x	x								x		x	x		x	x	x						
Tachyphonus cristatus	LC	LC	LC	x														x	x							
Tachyponus surinamus	LC	LC	LC	x														x	x							
Tachyphonus luctuosus	LC	LC	LC	x	x								x		x	x		x	x	x						
Tachyphonus delatrii	LC	LC	LC	x	x								x		x	x										
Tachyphonus rufus	LC	LC	LC	x	x								x		x	x		x	x	x						
Tachyphonus phoenicius	LC	LC	LC							x								x	x							
Lanio fulvus	LC	LC	LC	x	x										x			x	x							
Ramphocelus nigrogularis	LC	LC	LC	x														x	x							
Ramphocelus dimidiatus	LC	LC	LC	x	x								x		x	x				x						
Ramphocelus carbo	LC	LC	LC	x	x										x			x	x							
Ramphocelus flammigerus	LC	LC	LC	x	x								x		x	x										
Calochaetes coccineus	LC	LC	LC	x	x										x			x								
Bangsia arcaei	LC	LC	NT	x									x													
Bangsia melanochlamys	VU	VU	VU		x										x											
Bangsia rothschildi	LC	LC	LC	x	x								x		x											
Bangsia edwardsi	NT	NT	LC	x	x								x		x											
Bangsia aureocincta	VU	VU	EN		x										x											
Buthraupis montana	LC	LC	LC		x	x									x											
Buthraupis wetmorei	VU	VU	VU					x							x											
Cnemathraupis eximia	LC	LC	LC			x									x											
Chlorornis riefferii	LC	LC	LC		x	x									x											
Anisognathus melanogenys	LC	LC	LC		x	x														x						
Anisognathus lacrymosus	LC	LC	LC		x	x		x							x											
Anisognathus igniventris	LC	LC	LC		x	x									x											
Anisognathus somptuosus	LC	LC	LC		x	x									x											
Anisognathus notabilis	LC	LC	LC	x	x										x											
Dubusia taeniata	LC	LC	LC		x	x									x					x						
Iridosornis porphyrocephalus	LC	LC	NT		x										x											
Iridosornis analis	LC	LC	LC		x										x											
Iridosornis rufivertex	LC	LC	LC		x	x									x											
Pipraeidea melanonota	LC	LC	LC		x	x									x											
Chlorochrysa phoenicotis	LC	LC	LC	x	x								x		x											
Chlorochrysa calliparaea	LC	LC	LC	x	x										x			x								
Chlorochrysa nitidissima	VU	VU	VU		x										x											

Family and species	National category 2002	National category 2016	Global category 2018	ECOSYSTEMS L. rain-forest	Sub-A. forest	High A. forest	L. dry forest	Par-amo	Man-grove	Sav-anna	Fwt.	C.wt	REGIONS Pacific	P. Ocean	Andes	Caribbean	C. Sea	Amazon	Orinoquia	SNSM	Darién highlands	Sa&Pr.	GROUPS CONSERVATION CONCERN Game-birds	T. forest insectiv.	Large Frugiv.	Forest Raptors
Thraupis episcopus	LC	LC	LC	x	x	x							x		x	x		x	x	x	x					
Thraupis glaucocolpa	LC	LC	LC				x									x										
Thraupis palmarum	LC	LC	LC	x	x		x						x		x	x		x	x	x						
Thraupis cyanocephala	LC	LC	LC		x	x									x					x						
Tangara ruficervix	LC	LC	LC		x										x											
Tangara cyanoptera	LC	LC	LC	x	x										x					x						
Tangara heinei	LC	LC	LC		x										x					x						
Tangara palmeri	LC	LC	LC	x									x								x					
Tangara cayana	LC	LC	LC							x					x	x		x	x							
Tangara vitriolina	LC	LC	LC		x										x	x										
Tangara nigrocincta	LC	LC	LC	x														x	x							
Tangara larvata	LC	LC	LC	x	x								x		x	x										
Tangara cyanicollis	LC	LC	LC		x										x											
Ixothraupis rufigula	LC	LC	LC	x	x								x		x											
Ixothraupis guttata	LC	LC	LC	x	x										x						x					
Ixothraupis xanthogastra	LC	LC	LC	x	x										x			x								
Tangara vassorii	LC	LC	LC		x	x									x											
Tangara nigroviridis	LC	LC	LC		x	x									x											
Tangara fucosa	VU	VU	NT		x																x					
Tangara labradorides	LC	LC	LC		x	x									x											
Tangara cyanotis	LC	LC	LC		x										x											
Tangara inornata	LC	LC	LC	x	x								x		x	x										
Tangara mexicana	LC	LC	LC	x														x	x							
Tangara chilensis	LC	LC	LC	x	x													x	x							
Tangara velia	LC	LC	LC	x														x	x							
Tangara callophrys	LC	LC	LC	x														x								
Tangara lavinia	LC	LC	LC	x									x		x											
Tangara gyrola	LC	LC	LC	x	x										x			x	x	x	x					
Tangara chrysotis	LC	LC	LC		x										x											
Tangara xanthocephala	LC	LC	LC		x										x											
Tangara parzudakii	LC	LC	LC	x	x	x									x											
Tangara schrankii	LC	LC	LC	x	x										x			x								
Tangara johannae	LC	LC	NT	x									x													
Tangara arthus	LC	LC	LC		x										x											
Tangara florida	LC	LC	LC	x	x								x		x						x					
Tangara icterocephala	LC	LC	LC	x	x										x						x					
Tersina viridis	LC	LC	LC	x	x								x		x	x		x	x	x						
Dacnis albiventris	LC	LC	LC	x														x	x							
Dacnis lineata	LC	LC	LC	x	x								x		x	x		x	x							
Dacnis flaviventer	LC	LC	LC	x	x										x			x	x							
Dacnis hartlaubi	VU	VU	VU		x										x											
Dacnis venusta	LC	LC	LC	x									x			x					x					
Dacnis cayana	LC	LC	LC	x	x								x		x	x		x	x	x						
Dacnis viguieri	NT	NT	NT	x									x													
Dacnis berlepschi	VU	EN	VU	x	x										x											
Cyanerpes nitidus	LC	LC	LC	x														x	x							
Cyanerpes lucidus	LC	LC	LC	x									x													
Cyanerpes caeruleus	LC	LC	LC	x	x								x		x	x		x	x	x						
Cyanerpes cyaneus	LC	LC	LC	x									x		x	x		x		x						
Chlorophanes spiza	LC	LC	LC	x	x								x		x	x		x	x	x						
Iridophanes pulcherrimus	LC	LC	LC		x										x											
Heterospingus xanthopygius	LC	LC	LC	x	x								x		x	x										
Hemithraupis guira	LC	LC	LC	x	x		x						x		x	x		x	x							
Hemithraupis flavicollis	LC	LC	LC	x	x								x		x	x		x	x							
Chrysothlypis chrysomelas	LC	LC	LC	x	x																x					
Chrysothlypis salmoni	LC	LC	LC	x	x								x		x											
Conirostrum speciosum	LC	LC	LC	x			x											x	x							

Family and species	National category 2002	National category 2016	Global category 2018	ECOSYSTEMS									REGIONS										GROUPS CONSERVATION CONCERN			
				L. rain-forest	Sub-A. forest	High A. forest	L. dry forest	Par-amo	Man-grove	Sav-anna	Fwt.	C.wt	Pacific	P. Ocean	Andes	Caribbean	C. Sea	Amazon	Orinoquia	SNSM	Darién highlands	Sa&Pr.	Game-birds	T. forest insectiv.	Large Frugiv.	Forest Raptors
Conirostrum leucogenys	LC	LC	LC	x	x								x		x	x										
Conirostrum bicolor	LC	LC	NT						x		x						x	x								
Conirostrum cinereum	LC	LC	LC			x									x											
Conirostrum sitticolor	LC	LC	LC		x	x									x											
Conirostrum albifrons	LC	LC	LC		x	x									x											
Conirsotrum rufum	LC	LC	LC			x									x											
Diglossa gloriosissima	VU	VU	EN			x									x											
Diglossa lafresnayii	LC	LC	LC			x									x											
Diglossa humeralis	LC	LC	LC		x	x									x					x						
Diglossa brunneiventris	LC	LC	LC		x	x									x											
Diglossa albilatera	LC	LC	LC		x	x									x					x						
Diglossa indigotica	LC	LC	LC	x	x										x											
Diglossa sittoides	LC	LC	LC		x	x									x					x						
Diglossa glauca	LC	LC	LC		x										x											
Diglossa caerulescens	LC	LC	LC		x	x									x											
Diglossa cyanea	LC	LC	LC		x	x									x											
Catamblyrhynchus diadema	LC	LC	LC		x	x									x					x						
Urothraupis stolzmanni	LC	LC	LC			x									x											
Geospizopsis unicolor	LC	LC	LC					x							x					x						
Spodiornis rusticus	LC	LC	LC		x	x									x					x						
Sicalis citrina	LC	LC	LC							x					x				x							
Sicalis columbiana	LC	LC	LC							x									x							
Sicalis flaveola	LC	LC	LC							x			x		x	x		x	x	x						
Sicalis luteola	LC	LC	LC							x					x	x			x							
Emberizoides herbicola	LC	LC	LC							x					x	x			x	x						
Saltator maximus	LC	LC	LC	x	x								x		x	x		x	x	x	x					
Saltator atripennis	LC	LC	LC	x	x										x											
Saltator orenocensis	LC	LC	LC				x			x						x			x							
Saltator coerulescens	LC	LC	LC	x	x		x								x	x		x	x	x						
Saltator striatipectus	LC	LC	LC		x		x						x		x	x				x						
Saltator cinctus	VU	VU	NT		x	x									x											
Saltator grossus	LC	LC	LC	x	x								x		x	x		x	x							
Volatinia jacarina	LC	LC	LC		x		x			x			x		x	x		x	x	x	x					
Sporophila bouvronides	LC	LC	LC	x			x			x						x		x	x	x						
Sporophila lineola	LC	LC	LC							x						x		x	x							
Sporophila telasco	LC	LC	LC							x			x													
Sporophila castaneiventris	LC	LC	LC	x	x										x			x	x							
Sporophila minuta	LC	LC	LC							x			x		x	x		x	x	x						
Sporophila funerea	LC	LC	LC	x	x								x		x	x				x						
Sporophila angolensis	LC	LC	LC	x												x		x	x							
Sporophila crassirostris	LC	LC	LC	x	x										x	x		x	x							
Sporophila corvina	LC	LC	LC	x	x								x		x											
Sporophila intermedia	LC	LC	LC	x	x		x			x					x	x			x	x						
Sporophila murallae	LC	LC	LC	x														x								
Sporophila fringilloides	LC	LC	LC	x						x								x	x							
Sporophila luctuosa	LC	LC	LC		x										x					x						
Sporophila nigricollis	LC	LC	LC	x	x		x						x		x	x		x	x	x						
Sporophila caerulescens	LC	LC	LC	x														x								
Sporophila schistacea	LC	LC	LC	x	x								x		x	x			x							
Sporophila plumbea	LC	LC	LC							x					x	x			x							
Catamenia analis	LC	LC	LC					x							x					x						
Catamenia inornata	LC	LC	LC					x		x					x											
Catamenia homochroa	LC	LC	LC			x		x							x					x						
Coryphospingus pileatus	LC	LC	LC				x									x				x						
Coereba flaveola	LC	LC	LC	x	x				x				x	x	x	x	x	x	x	x	x	x				
Tiaris olivaceus	LC	LC	LC		x										x											
Tiaris obscurus	LC	LC	LC	x	x										x	x				x						

				ECOSYSTEMS									REGIONS										GROUPS CONSERVATION CONCERN			
**Family and species**	**National category 2002**	**National category 2016**	**Global category 2018**	**L. rain-forest**	**Sub-A. forest**	**High A. forest**	**L. dry forest**	**Par-amo**	**Man-grove**	**Sav-anna**	**Fwt.**	**C.wt**	**Pacific**	**P. Ocean**	**Andes**	**Caribbean**	**C. Sea**	**Amazon**	**Orinoquia**	**SNSM**	**Darién highlands**	**Sa&Pr.**	**Game-birds**	**T. forest insectiv.**	**Large Frugiv.**	**Forest Raptors**
Tiaris fuliginosus	LC	LC	LC		x		x								x					x						
Tiaris bicolor	LC	LC	LC		x		x			x					x	x						x				
Parkerthraustes humeralis	LC	LC	LC	x														x								
**Incertae Sedis**																										
Mitrospingus cassinii	LC	LC	LC	x	x								x		x	x										
Rhodinocichla rosea	LC	LC	LC		x		x								x					x						
**Emberizidae**																										
Oreothraupis arremonops	LC	LC	LC		x										x											
Chlorospingus flavigularis	LC	LC	LC	x	x										x											
Chlorospingus parvirostris	LC	LC	LC		x										x											
Chlorospingus canigularis	LC	LC	LC		x										x											
Chlorospingus flavopectus	LC	LC	LC		x	x									x											
Chlorospingus tacarcunae	NT	NT	LC		x																x					
Chlorospingus inornatus	VU	VU	LC		x																x					
Chlorospingus semifuscus	LC	LC	LC		x										x											
Chlorospingus flavovirens	VU	VU	N/A*		x								x													
Ammodramus savannarum	EN	EN	LC							x					x											
Ammnodramus humeralis	LC	LC	LC							x					x	x			x	x						
Ammnodramus aurifrons	LC	LC	LC	x	x										x	x		x	x							
Arremonops conirostris	LC	LC	LC	x	x		x						x		x	x		x	x	x						
Arremonops tocuyensis	NT	NT	LC				x									x										
Arremon basilicus	LC	LC	NT		x	x														x						
Arremon perijanus	LC	LC	VU		x	x									x											
Arremon atricapillus	LC	LC	LC	x	x								x		x						x					
Arremon assimilis	LC	LC	LC		x	x									x											
Arremon aurantiirostris	LC	LC	LC		x	x							x		x	x										
Arremon schlegeli	VU	VU	LC		x		x								x	x				x						
Arremon taciturnus	LC	LC	LC	x														x	x							
Arremon brunneinucha	LC	LC	LC		x	x									x						x					
Arremon castaneiceps	LC	LC	NT		x										x											
Zonotrichia capensis	LC	LC	LC	x	x	x									x	x		x	x	x						
Atlapetes albinucha	LC	LC	LC		x										x											
Atlapetes albofrenatus	LC	LC	LC		x	x									x											
Atlapetes melanocephalus	LC	LC	LC		x	x														x						
Atlapetes semirufus	LC	LC	LC		x	x									x											
Atlapetes flaviceps	VU	VU	EN		x										x											
Atlapetes fuscoolivaceus	VU	VU	NT		x										x											
Atlapetes leucopis	LC	LC	LC		x	x									x											
Atlapetes tricolor	LC	LC	LC	x	x										x											
Atlapetes schistaceus	LC	LC	LC		x	x									x											
Atlapetes pallidinucha	LC	LC	LC			x									x											
Atlapetes blancae	CR	CR	CR			x									x											
Atlapetes latinuchus	LC	LC	LC		x	x									x											
**Cardinalidae**																										
Piranga flava	LC	LC	LC		x										x					x						
Piranga rubra	LC	LC	LC	x	x	x	x						x		x	x		x	x	x	x	x				
Piranga olivacea	LC	LC	LC	x	x	x							x		x	x		x	x	x	x	x				
Piranga rubriceps	LC	LC	LC		x	x									x											
Piranga leucoptera	LC	LC	LC		x										x											
Habia rubica	LC	LC	LC	x											x			x								
Habia fuscicauda	LC	LC	LC				x									x										
Habia gutturalis	LC	LC	NT	x	x										x	x										
Habia cristata	LC	LC	LC	x	x										x											
Chlorothraupis carmioli	LC	LC	LC	x	x										x			x			x					
Chlorothraupis olivacea	LC	LC	LC	x									x													
Chlorothraupis stolzmanni	LC	LC	LC	x	x										x											
Pheucticus chrysogaster	LC	LC	LC		x	x									x					x						

Family and species	National category 2002	National category 2016	Global category 2018	ECOSYSTEMS									REGIONS										GROUPS CONSERVATION CONCERN			
				L. rain-forest	Sub-A. forest	High A. forest	L. dry forest	Par-amo	Man-grove	Sav-anna	Fwt.	C.wt	Pacific	P. Ocean	Andes	Caribbean	C. Sea	Amazon	Orinoquia	SNSM	Darién highlands	Sa&Pr.	Game-birds	T. forest insectiv.	Large Frugiv.	Forest Raptors
Pheucticus aureoventris	LC	LC	LC		x	x									x											
Pheucticus ludovicianus	LC	LC	LC			x							x		x	x				x	x	x				
Granatellus pelzelni	LC	LC	LC	x														x	x							
Cardinalis phoeniceus	VU	VU	LC				x									x										
Caryothraustes canadensis	LC	LC	LC	x														x	x		x					
Amaurospiza concolor	LC	LC	LC		x										x											
Cyanoloxia cyanoides	LC	LC	LC	x	x								x		x	x		x	x	x						
Cyanoloxia brissonii	LC	LC	LC		x		x								x	x										
Spiza americana	LC	LC	LC							x			x		x	x			x	x		x				
**Parulidae**																										
Parkesia noveboracensis	LC	LC	LC	x	x	x			x				x	x	x	x	x	x	x	x	x	x		x		
Parkesia motacilla	LC	LC	LC	x	x										x	x				x				x		
Vermivora chrysoptera	LC	LC	NT	x	x								x		x	x				x	x	x				
Mniotilta varia	LC	LC	LC	x	x	x							x		x	x				x	x	x				
Protonotaria citrea	LC	LC	LC	x	x		x		x				x	x	x	x	x			x	x	x				
Leiothlypis peregrina	LC	LC	LC	x	x	x							x		x	x				x	x	x				
Oporornis agilis	LC	LC	LC	x			x								x	x		x	x			x				
Geothlypis aequinoctialis	LC	LC	LC	x							x					x		x	x							
Geothlypis philadelphia	LC	LC	LC	x	x	x	x						x		x	x				x	x	x				
Geothlypis formosa	LC	LC	LC	x	x		x						x		x	x				x	x	x				
Geothlypis semiflava	LC	LC	LC	x							x		x		x	x										
Setophaga ruticilla	LC	LC	LC	x	x		x						x		x	x		x	x	x	x	x				
Setophaga cerulea	VU	VU	VU	x	x										x					x						
Setophaga pitiayumi	LC	LC	LC	x	x	x							x		x	x				x	x					
Setophaga castanea	LC	LC	LC	x	x		x						x		x	x				x		x				
Setophaga fusca	LC	LC	LC	x	x	x							x		x	x			x	x	x	x				
Setophaga petechia	LC	LC	LC	x	x	x			x				x	x	x	x	x	x	x	x		x				
Setophaga pensylvanica	LC	LC	LC	x									x		x	x				x		x				
Setophaga striata	LC	LC	NT	x									x		x	x		x	x	x		x				
Myiothlypis luteoviridis	LC	LC	LC		x	x									x											
Myiothlypis basilica	EN	EN	VU		x	x														x						
Myiothlypis flaveola	LC	LC	LC				x								x	x										
Myiothlypis nigrocristata	LC	LC	LC		x	x									x											
Myiothlypis fulvicauda	LC	LC	LC								x		x		x	x		x								
Myiothlypis chrysogaster	LC	LC	LC	x	x										x											
Myiothlypis conspicillata	VU	VU	NT		x															x						
Myiothlypis cinereicollis	NT	NT	NT		x										x											
Myiothlypis coronata	LC	LC	LC		x	x									x											
Basileuterus rufifrons	LC	LC	LC	x	x										x	x				x						
Basileuterus culicivorus	LC	LC	LC	x	x										x					x						
Basileuterus ignotus	EN	EN	VU		x																x					
Basileuterus tristriatus	LC	LC	LC	x	x	x									x						x					
Cardellina canadensis	LC	LC	LC	x	x	x							x		x	x				x	x	x				
Myioborus miniatus	LC	LC	LC	x	x	x									x					x	x					
Myioborus flavivertex	LC	LC	LC		x	x														x						
Myioborus ornatus	LC	LC	LC		x	x									x											
Myioborus melanocephalus	LC	LC	LC		x	x									x											
**Icteridae**																										
Psarocolius angustifrons	LC	LC	LC	x	x	x									x			x	x						x	
Psarocolius viridis	LC	LC	LC	x														x	x						x	
Psarocolius wagleri	LC	LC	LC	x	x								x		x	x									x	
Psarocolius decumanus	LC	LC	LC	x	x								x		x	x		x	x	x					x	
Psarocolius guatimozinus	LC	LC	LC	x									x			x									x	
Psarocolius cassini	EN	EN	EN	x									x												x	
Psarocolius bifasciatus	LC	LC	LC	x														x	x						x	
Cacicus solitarius	LC	LC	LC	x														x	x							
Cacicus sclateri	LC	LC	LC	x														x								

Family and species	National category 2002	National category 2016	Global category 2018	ECOSYSTEMS									REGIONS										GROUPS CONSERVATION CONCERN			
				L. rain-forest	Sub-A. forest	High A. forest	L. dry forest	Par-amo	Man-grove	Sav-anna	Fwt.	C.wt	Pacific	P. Ocean	Andes	Caribbean	C. Sea	Amazon	Orinoquia	SNSM	Darién highlands	Sa&Pr.	Game-birds	T. forest insectiv.	Large Frugiv.	Forest Raptors
Cacicus uropygialis	DD	DD	LC		x										x											
Cacicus cela	LC	LC	LC	x	x								x		x	x		x	x	x					x	
Cacicus chrysonotus	LC	LC	LC		x	x									x										x	
Cacicus latirostris	LC	LC	LC	x														x							x	
Cacicus haemorrhous	LC	LC	LC	x														x	x						x	
Cacicus oseryi	LC	LC	LC	x														x							x	
Amblyercus holosericeus	LC	LC	LC	x	x	x									x					x	x					
Icterus icterus	VU	VU	LC				x			x						x			x							
Icterus croconotus	LC	LC	LC	x														x								
Icterus mesomelas	LC	LC	LC	x	x								x		x	x				x						
Icterus cayanensis	LC	LC	LC	x	x										x			x	x							
Icterus spurius	LC	LC	LC	x			x						x		x	x				x	x					
Icterus auricapillus	LC	LC	LC	x	x		x						x		x	x			x	x						
Icterus chrysater	LC	LC	LC	x	x	x							x		x	x				x						
Icterus galbula	LC	LC	LC	x	x		x						x		x	x				x	x	x				
Icterus leucopteryx	CR	CR	LC				x															x				
Icterus nigrogularis	LC	LC	LC		x		x								x	x			x	x						
Macroagelaius subalaris	EN	EN	EN		x	x									x											
Gymnomystax mexicanus	LC	LC	LC								x					x		x	x							
Hypopyrrhus pyrohypogaster	VU	VU	VU		x										x											
Lampropsar tanagrinus	LC	LC	LC	x							x							x	x							
Chrysomus icterocephalus	LC	LC	LC								x				x	x		x	x							
Molothrus oryzivorus	LC	LC	LC	x	x		x			x			x		x	x		x	x	x					x	
Molothrus aeneus	VU	VU	VU				x									x										
Molothrus bonariensis	LC	LC	LC	x	x	x	x			x			x		x	x		x	x	x						
Quiscalus lugubris	LC	LC	LC							x					x	x			x	x						
Quiscalus mexicanus	LC	LC	LC						x			x		x			x								x	
Sturnella magna	LC	LC	NT					x		x					x	x			x	x						
Sturnella militaris	LC	LC	LC	x	x		x			x			x		x	x		x	x							
Sturnella bellicosa	LC	LC	LC							x			x													
**Fringillidae**																										
Spinus spinescens	LC	LC	LC		x	x		x							x					x						
Spinus cucullatus	EN	EN	EN				x			x						x										
Spinus magellanicus	LC	LC	LC		x	x									x											
Spinus xanthogastrus	LC	LC	LC		x	x									x											
Spinus psaltria	LC	LC	LC	x	x	x									x	x				x						
Euphonia plumbea	LC	LC	LC	x														x	x							
Euphonia chlorotica	LC	LC	LC	x														x	x							
Euphonia trinitatis	LC	LC	LC				x									x										
Euphonia concinna	LC	LC	LC		x		x								x	x										
Euphonia saturata	LC	LC	LC	x	x		x						x		x											
Euphonia laniirostris	LC	LC	LC		x		x								x	x		x	x	x						
Euphonia cyanocephala	LC	LC	LC		x	x									x					x						
Euphonia fulvicrissa	LC	LC	LC	x									x		x	x										
Euphonia chrysopasta	LC	LC	LC	x	x										x			x	x							
Euphonia mesochrysa	LC	LC	LC	x	x										x											
Euphonia minuta	LC	LC	LC	x									x			x		x								
Euphonia anneae	LC	LC	LC	x	x																x					
Euphonia xanthogaster	LC	LC	LC	x	x	x							x		x	x		x	x		x					
Euphonia rufiventris	LC	LC	LC	x														x	x							
Chlorophonia cyanea	LC	LC	LC	x	x										x					x						
Chlorophonia pyrrhoprhys	LC	LC	LC		x	x									x											
Chlorophonia flavirostris	LC	LC	LC	x	x										x						x					

N/A* These species are absent in the Birdlife database. They are either a subspecies of another taxon, or a species recently described by science.
