# Supplementary material for: Tracking extinction risk trends and patterns in a mega-diverse country: A Red List Index for birds in Colombia
Source: PLoS One. 2020 Jan 27;15(1):e0227381. doi: 10.1371/journal.pone.0227381 (PMC6984723; doi:10.1371/journal.pone.0227381)
Supplement: Attachment — Submitted filename: Response to Reviewers.docx [file pone.0227381.s003.docx]

**Reviewer #1:**

First of all, we would like to thank your detailed and helpful comments. They have contributed to improve our paper in an important way.

Dear authors Brazil has the richest avifauna in the world (see Piacentini et al. 2015), with 1.919 species. Please change this information throughout the manuscript.

*Response:* Differences in species numbers between different sources are the result of both species diversity and the taxonomic philosophy underlying each species list. However, comparing works using the same taxonomic approach (BirdLife International <http://www.birdlife.org/datazone/country/colombia> and South America Classification Committee <http://www.museum.lsu.edu/~Remsen/SACCBaseline.htm>), Brazil comes in third place at a global scale after Colombia and Peru. In fact, Piacentini et al. 2015 state that: “Forming almost half of the “Bird Continent” of South America, Brazil vies for the title of the country with the richest avifauna along with Colombia and Peru (the latest statistics put it in second, after Colombia; Remsen *et al.* 2015).” Appropriate references have therefore been inserted in the text to justify this statement (see lines 122-124).

Please change the Y-axis of the graphs - it is more intuitive for the reader to perceive the increase of the extinction risk looking up, to the tip of the arrow. As shown in the ms it is not very intuitive and the output of the graphs must be changed.

*Response:* The Red List Index is widely used and recognized now, being published in dozens of papers, used by the CBD to track progress to the Aichi targets (<https://www.cbd.int/gbo4/>), adopted by the United Nations to track progress to the Sustainable Development Goals (<https://unstats.un.org/sdgs/indicators/database/>), and included in many assessments and reports and policy processes (e.g. the [Global Assessment of the Intergovernmental Science-Policy Platform on Biodiversity and Ecosystem Services](https://ipbes.net/news/ipbes-global-assessment-summary-policymakers-pdf)). The index was specifically formulated so that the graph goes down as things get worse, to be consistent with most other biodiversity indicators (eg the Living Planet Index, Wild Bird Index, Biodiversity Intactness Index etc). We therefore prefer to retain its current format for consistency and to avoid confusion. However, we added new text to explain this specifically. (See lines 181-183).

The results are well presented, clear and concise. However, the discussion should be improved and supported by more data or references (e.g. There are no protected areas within the Darién Highlands in Colombia, and there is very limited protection within San Andrés and Providencia, lacking a reference or vague information - what's very limited?).

*Response:* We have edited the sentence to improve clarity and added a reference (see lines 397-399): “Los Katíos National Park covers only part of the Darien lowlands, but no highland species are protected within it, and there is only one small terrestrial protected area on each of San Andrés and Providencia islands (RUNAP 2019).”

Another example: "again suggesting that, on average, the level of threat to birds in Colombia is worsening less rapidly than at a global scale.", needs a reference or a more precise comparison.

*Response:* We have provided the precise numeric values for decline rates and inserted the appropriate reference in line 409 for the global index.

The discussion needs to be improved. The section "Threats to birds in Colombia" is very important, and should be improved with scientific data - some parts of the ms are lacking data which could be obtained quite easily (e.g. Agricultural expansion, including for cattle ranching, affected two uplisted species, both in Orinoquia: Bearded Tachuri (*Polystictus pectoralis*) and Orinoco Goose (*Oressochen jubatus*).) How? These species are extremely distinct in habitat requirements and behavior and other natural history traits, and, despite briefly described in the table, need to be better detailed. Orinoco Goose is suffering from the expansion of rice fields? What's the real threat for this species?

*Response:* We have included further detail on factors affecting both species see lines 494-502: Fire, affecting almost the entire Orinoquia, and agricultural expansion, including for cattle ranching, affected two uplisted species, both in Orinoquia: Bearded Tachuri (*Polystictus pectoralis*) and Orinoco Goose (*Oressochen jubatus*). The former depends on natural grasslands and is affected by an increase in the frequency of fires for livestock grazing, and more locally by habitat transformation for agriculture. The subspecies *P. pectoralis bogotensis* has already been driven extinct by habitat transformation in the Eastern Andes and the dry valleys in the west of the country [42]. Orinoco Goose depends on seasonally flooded savannas, wetlands, and forest for feeding and nesting, habitats that are all being affected by the expansion of agriculture and intensive cattle-raising [90].

The ms is well-written, lacking few adjustments to be published.

**Reviewer #2:**

First of all, the authors would like to thank your detailed and helpful comments. They have contributed to improve our paper in an important way.

I find one important thing missing in the paper. There should be a supplementary table with all 1718 species that the authors analyzed showing each species, its current category, the category at 2002, the global category, the region, the ecosystem, and the species group to which it was assigned. This is needed for readers to see the transparency of the study and be able to replicate it.

*Response*: Absolutely. A supplementary table has been included as S2.

There is another addition that would make the paper a lot stronger, and it is the reason why I suggest major revisions. You discuss the importance of some groups in driving the national RLI, but you have no statistical test to back this up. I suggest adding group testing statistical tests, like ANOVA for each of your categories, to see which one is driving the index (statistically). This would really show which group of species, ecosystem, or region is driving the national total. Once this is incorporated to the methods, results, and discussion, the paper will be ready for a second revision and then publication.

*Response*: this is a good point but it is important to consider in relation to the nature of the data. The key point is to partition the proportion of variance due to different factors. However, our data are categorical and not continuous (regions, ecosystems and species groups of conservation concern). Further, our data are counts (numbers of species in each region, ecosystem or group, and in each IUCN category, etc.), and they are not entirely independent from each other. For instance, species can be found in one or several regions. Perhaps more importantly, risk assessments were conducted at a national level, lumping all regions together. Therefore, although this sort of analysis would have been highly desirable, the data do not permit it. However, we identified statements in the paper that required further statistical analysis to justify them. We therefore performed additional contingency tables analyses, following the specific suggestion by the reviewer in order to determine which regions are statistically driving the Red List Index (RLI), and used contingency table analysis when expected numbers in the contingency tables were greater than 5 (see lines 305-309).

Here are some detailed comments:

Line 28: it would be useful to explain in a short way what a higher index means. Higher index, more threat? This is key because then you go on to explaining your results and the reader might not know about the RLI.

*Response:* Good point. We have added text to clarify this. See the current lines 28-29.

Line 35: maybe it should read “…are the most threatened”, or “…For x, y, ans z the threat status has worsened/the extinction risk increased”

*Response:* Done. See the current line 36.

Line 36: invasive animals, or species? Invasive plants are often also involved in declining populations of birds.

*Response:* We meant animals and have edited the text to clarify this. See the current line 38.

Line 54: check the use of the word “it” in “it consumes”

*Response:* Done. See the current line 59.

Lines 77-80: check sentence and rephrase to make more clear

*Response:* We have reworded this sentence. See the current lines 85-87.

Line 83: a “sampled approach”..what does that mean? Using field data? Ground trothing? Please clarify.

*Response:* We have provided additional text to clarify this, (see lines 90-93): The sampled approach involves assessing random selection of species from across the entire taxonomic group, allowing for the assessment of trends for large taxonomic groups [Baillie et al. 2008].

Line 85-86: change to “where conservation resources are also typically allocated”

*Response:* Done. See the current line 95-96.

Line 104-106: this reads more like a discussion that part of the introduction. Leave it out here and include it in the discussion of your results.

*Response:* We prefer to keep this sentence here to give a context to our study in terms of its relevance as well as previous studies leading to this paper.

Line 118: it is not clear which are “these sources”. Do you mean the extinction assessments mentioned above? Make this clear.

*Response:* We have edited to text to make this clear. See current line 141.

Line 145-151: a map delineating these regions would be a really nice addition to the paper. I suggest including it.

*Response:* We agree. Please see the current line 201 and the new figure 1.

Line 158-159: why was the Darien treated differently? Explain and maybe include a source.

*Response:* We have now provided an explanation: This is because on small isolated mountains the upper limit of lowland rain forest is lower than on major ranges due to the “Massenerhebung” effect (Grubb 1971). See current lines 208-210.

Lines 160-170: this would be easier to read in a table. Also I strongly advise including a supplementary table with how you classified each species in each region, ecosystem, and species group so readers can track what is happening with each species, and so they can replicate the study.

*Response:* This point is addressed with the inclusion of the new supplementary table (S2), as it includes the whole database with the classifications of each species in each region, group and other classes.

Line 204-206: Does this mean you can only use this 18% in your analyses. You should clarify that.

*Response:* The calculation of the RLI uses the complete list of species and their current red list categories, but trends since the first time-point are driven only by the changes in categories for the 18% of species for which these were genuine. We consider have added text to clarify this.:” These category changes drive trends in the RLI, although the full set of species and their current red list categories are used to determine the 2016 RLI value.” See lines 271-273.

Lines 201-222: Only cite Table 2 once. Currently you cite it like 4 times in the paragraph.

*Response:* Done. See page 10.

Lines 229-237: I would suggest using a statistical test that compares differences between groups, to see if different ecosystems, regions, or species groups had significant impact on the overall change of RLI. Maybe an ANOVA.

*Response*: Please see second response to reviewer 2.

Table 2: Orinoco Goose: you mention forest loss as important for this species, it probably is due to their nesting grounds, but not so much their every-day life. Please specify that forest is needed for nesting, I think this would help the reader understand the species situation better.

*Response:* Yes, we agree. We specify in table 2 (see the beginning of the table at line 328), that forest is needed for nesting.

In Black Inca: should be “mature” not “madure”

In Micrastur: “over the next…” and “illegal coca crops”

In Pipreola:” moved to other regions”

In Cistothorus: rephrase last sentence of the explanation. It should read better.

*Response:* all these minor changes were done in table 2.

Lines 252-255: this claim could use a reference to back it up.

*Response:* This is our finding and interpretation. We have edited the wording to clarify this (see current lines 368-369).

Lines 259-260: I would describe what happens in the Andes as deforestation, and not logging. Logging can be low impact, or selected (like they do in the Amazon), but the Andes have mostly been deforested for agriculture, pasture, or urbanization.

*Response:* we changed forest logging to “deforestation”. See current line 373.

Lines 261-262: Again, a statistical analysis testing the importance of groups is needed, and one to highlight which group has the strongest impact.

*Response:* We agree, so we performed new G-tests (see lines 231-233 also 306-309.

Lines 263-266: As you mentioned in the abstract, the peace process has played its role. Most of the Amazon deforestation is very recent and has happened after the signing of the peace treaty. Check out information by the project MAAP (<https://maaproject.org/en/> ) to get some updated deforestation statistics. Or IDEAM statistics would also be good to cite here.

*Response:* Thank you for the suggestion. We cited both, MAAP and Ideam 2019 (See current line 382).

Line 279: “less rapidly” should be changed to “slower”, “at a slower pace”, or something similar.

*Response:* Done. See line 411

Line 318: need a comma before “at least for threatened species”

*Response:* Done. See line 458

Lines 348-350: are oil palm plantations in savannas really that large to have a noticeable impact? Or are you referring more to the potential impact they could have. As I understand it, these plantations are very few and far between still (in savannas).

*Response:* This needed to be written more clearly. We edited this, and removed a reference oil palm: “Fire, affecting almost the entire Orinoquia, and agricultural expansion, including for cattle ranching, affected two uplisted species, both in Orinoquia: Bearded Tachuri (*Polystictus pectoralis*) and Orinoco Goose (*Oressochen jubatus*). The former depends on natural grasslands and is affected by an increase in the frequency of fires for livestock grazing, and more locally by habitat transformation for agriculture. The subspecies *P. pectoralis bogotensis* has already been driven extinct in by the habitat transformation in the Eastern Andes and the dry valleys in the west of the country [42]. Orinoco Goose depends on seasonally flooded savannas, wetlands, and forest for feeding and nesting, habitats that are all being affected by the expansion of agriculture and intensive cattle-raising [90].” Lines 495-503.

Lines 363-364: fins a reference to back up this statement. There are social scientists studying subsistence hunting and papers are out there.

*Response:* We added two appropiate references regarding this point (Robinson and Redford 1991, Sarti et al 2015). See current line 528.

References: check ALL your references for style. I found some mistakes but did not check all the references. Please make sure they are all correct before resubmitting your manuscript.

*Response:* We checked again all references to correct a few mistakes.
